# Therapeutic KRAS^G12C^ inhibition drives effective interferon-mediated antitumor immunity in immunogenic lung cancers

**DOI:** 10.1126/sciadv.abm8780

**Published:** 2022-07-20

**Authors:** Edurne Mugarza, Febe van Maldegem, Jesse Boumelha, Christopher Moore, Sareena Rana, Miriam Llorian Sopena, Philip East, Rachel Ambler, Panayiotis Anastasiou, Pablo Romero-Clavijo, Karishma Valand, Megan Cole, Miriam Molina-Arcas, Julian Downward

**Affiliations:** ^1^ Oncogene Biology Laboratory, Francis Crick Institute, 1 Midland Road, London NW1 1AT, UK.; ^2^ Bioinformatics and Biostatistics Science Technology Platform, Francis Crick Institute, 1 Midland Road, London NW1 1AT, UK.; ^3^ Lung Cancer Group, Division of Molecular Pathology, Institute of Cancer Research, 237 Fulham Road, London SW3 6JB, UK.

## Abstract

Recently developed KRAS^G12C^ inhibitory drugs are beneficial to lung cancer patients harboring KRAS^G12C^ mutations, but drug resistance frequently develops. Because of the immunosuppressive nature of the signaling network controlled by oncogenic KRAS, these drugs can indirectly affect antitumor immunity, providing a rationale for their combination with immune checkpoint blockade. In this study, we have characterized how KRAS^G12C^ inhibition reverses immunosuppression driven by oncogenic KRAS in a number of preclinical lung cancer models with varying levels of immunogenicity. Mechanistically, KRAS^G12C^ inhibition up-regulates interferon signaling via Myc inhibition, leading to reduced tumor infiltration by immunosuppressive cells, enhanced infiltration and activation of cytotoxic T cells, and increased antigen presentation. However, the combination of KRAS^G12C^ inhibitors with immune checkpoint blockade only provides synergistic benefit in the most immunogenic tumor model. KRAS^G12C^ inhibition fails to sensitize cold tumors to immunotherapy, with implications for the design of clinical trials combining KRAS^G12C^ inhibitors with anti-PD1 drugs.

## Teaser

KRAS inhibition mobilizes antitumor immunity in immunogenic lung cancer models through derepressing interferon signaling.

## INTRODUCTION

Lung cancer is the number one cause of cancer deaths worldwide, leading to some 1.8 million deaths annually, and therefore represents a disease of very high unmet need (*1*). Non–small cell lung cancer (NSCLC) comprises 84% of all lung cancers and has a 5-year survival rate of only 25% (*2*). Fortunately, with the introduction of immune checkpoint blockade (ICB), such as anti-PD1 therapy, aiming to boost antitumor T cell immunity, the paradigm for treatment has shifted, enabling long-lasting responses in a subset of patients (*3*). However, only a minority of patients respond and, of those that do, many develop resistance to treatment over time; hence, great efforts are currently aimed at trialing therapeutic combinations with ICB (*4*). Targeting oncogenic drivers has been another approach to control tumor growth, as recurrent genetic alterations are detected in more than half of lung adenocarcinoma patients (*5*). Targeted inhibition of receptor tyrosine kinases such as epidermal growth factor receptor (EGFR) has extended progression-free survival beyond conventional cytotoxic therapies. But, until recently, inhibiting KRAS, the most frequent target of oncogenic mutations found in about 15% of all cancer patients and 33% of those with lung adenocarcinoma (*6*), has been notoriously difficult. In 2013, Ostrem *et al.* (*7*) reported the development of a covalent inhibitor that was able to lock KRAS into its inactive guanosine diphosphate (GDP)–bound state by binding to the cysteine resulting from the G12C mutation, present in 40% of KRAS-mutant NSCLC patients. A mutation-specific inhibitor would be able to circumvent the high toxicity that has limited the widespread use of compounds targeting signaling downstream of KRAS, such as mitogen-activated protein kinase (MAPK) kinase (MEK) inhibitors. The discovery of a KRAS^G12C^-specific compound led to rapid development of clinical inhibitors, and in 2021, Amgen was the first to obtain U.S. Food and Drug Administration approval for clinical use of AMG510 (sotorasib) in locally advanced or metastatic KRAS^G12C^-mutant NSCLC (*8*–*10*). As expected, toxicity from these drugs is low as signaling is only inhibited in cancer cells harboring the G12C mutation, and clinical response rates are high, but unfortunately, resistance occurs frequently within a few months of treatment. Several mechanisms of resistance have already been described, including alternative mutations in KRAS or bypassing the mutation via redundant signaling pathways (*11*–*13*). Hence, combination therapies will be needed to make a greater impact on patient survival (*14*, *15*).

Exploring the combination of targeted inhibition of KRAS with anti-PD1 therapy seems an obvious approach, and the first clinical trials are already well underway. There are certainly rational arguments to make for this combination, with KRAS-mutant lung cancer generally being associated with a smoking history and therefore high tumor mutation burden, one of the positive predictors for response to ICB (*16*, *17*). Moreover, KRAS-mutant lung cancer is strongly associated with an immune evasive phenotype and KRAS signaling is thought to play a role in orchestrating such an immunosuppressive environment, for example, by driving the expression of cytokines and chemokines as was shown for interleukin-10 (IL-10), transforming growth factor–β (TGF-β), and granulocyte-macrophage colony-stimulating factor (GM-CSF) in KRAS-mutant pancreatic cancer (*18*). Inhibition of KRAS could provide temporary relief from this immunosuppression and a window of opportunity for T cell activation. Initial reports of KRAS^G12C^ inhibition showed that durable responses in mice were dependent on T cells, and a combination of KRAS^G12C^ inhibition and anti-PD1 led to improved survival in a subcutaneous tumor model of the genetically engineered G12C KRAS-mutant CT26 colon cancer cell line (*8*, *19*). While the KRAS^G12C^ mutation is only found in 3 to 4% of colon carcinoma, it is more prevalent in NSCLC (~14%) and clinical efficacy of the KRAS inhibitors also seems to be higher in lung cancer. Therefore, it will be most relevant to understand the mechanisms underlying potential therapeutic cooperation between KRAS inhibition and immune responses in the setting of lung cancer (*9*). Tissue site, existing immune evasive tumor microenvironment (TME), and intrinsic immunogenicity in the form of neoantigen presentation are all likely to be important factors in determining the outcome of combination treatments with ICB (*4*). Fedele *et al.* (*20*) showed that a combination of SHP2 and KRAS^G12C^ inhibition led to good tumor control and increased T cell infiltration in an orthotopic model of lung cancer. Using the strongly immune evasive 3LL ΔNRAS lung cancer cell line (*14*), we recently developed an imaging mass cytometry (IMC) analysis pipeline that showed that KRAS^G12C^ inhibition was able to induce remodeling of the lung TME (*21*). Here, we use this 3LL ΔNRAS alongside other preclinical lung cancer models varying in degree of immunogenicity to perform an in-depth investigation of the impact of KRAS^G12C^ inhibition on the TME and antitumor immunity and explore the mechanisms that underlie the changes observed. We describe several mechanisms by which tumor-specific KRAS inhibition has direct and indirect effects on the TME, such as reduced expression of chemokines attracting immunosuppressive myeloid cells, enhanced uptake of tumor cells by antigen-presenting cells (APCs), and enhanced intrinsic and extrinsic interferon (IFN) responses. Furthermore, we show that successful combination of KRAS^G12C^ inhibition with ICB is not universal, but rather varies between the models and correlates with immunogenicity, which will have important implications for the selection of patients who may benefit from this combination therapy. In particular, tumors that are refractory to ICB, due to either intrinsic or acquired resistance, may be unlikely to be resensitized by combination with KRAS^G12C^ inhibition alone.

## RESULTS

### Oncogenic KRAS regulates expression of cytokine and immune regulatory genes in human and murine cell lines

**Fig. 1.**
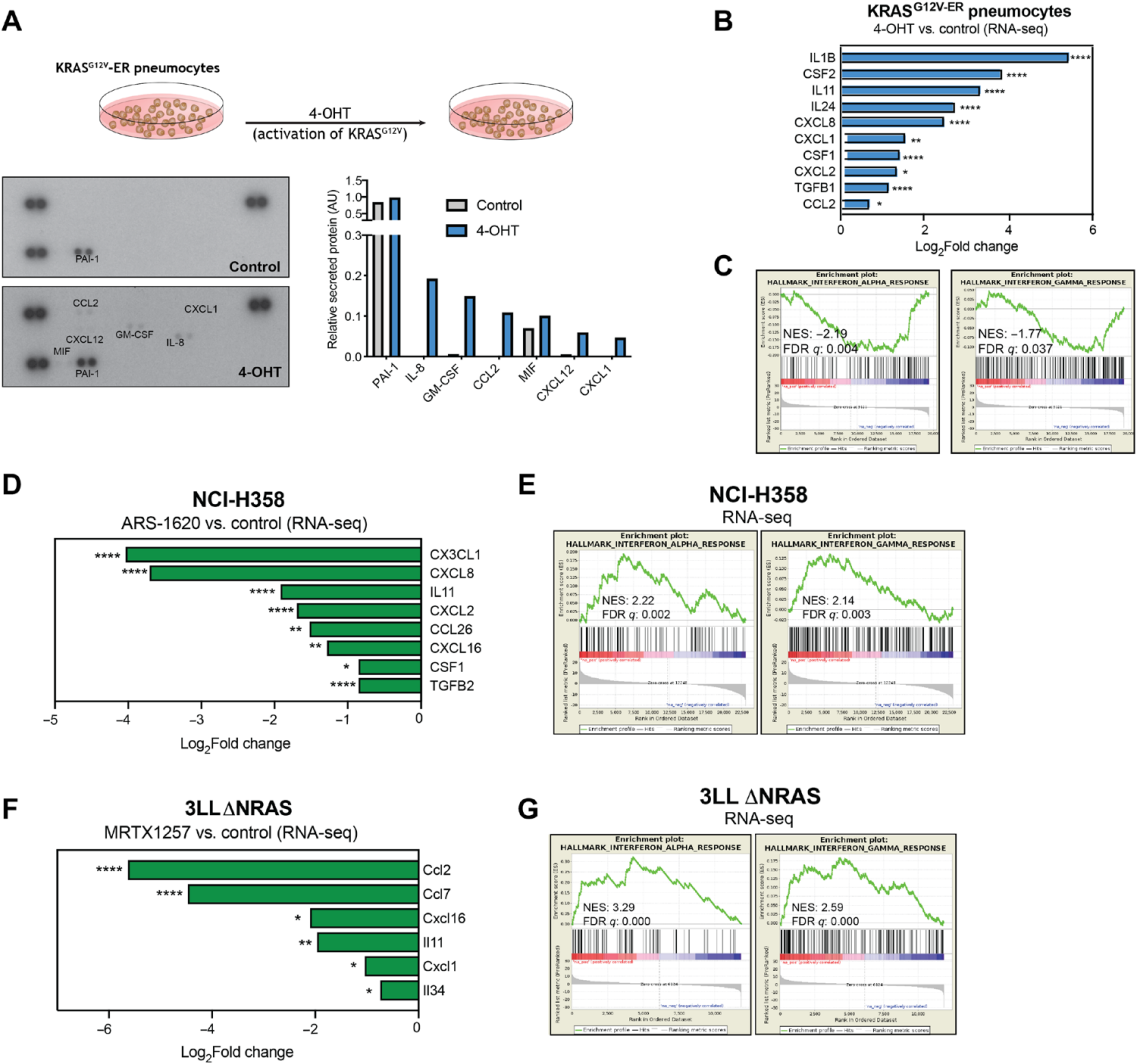
Oncogenic KRAS regulates immune gene expression in cell lines. (**A**) Cytokine array of cell culture supernatant from KRAS^G12V-ER^ pneumocytes treated with 500 nM 4-OHT or ethanol control for 24 hours. Graph shows secreted protein relative to control spots on the array for each condition. (**B**) Log_2_Fold change of selected cytokine genes from RNA-seq data in 4-OHT (500 nM, 24 hours)–treated KRAS^G12V-ER^ pneumocytes. (**C**) MSigDB Hallmarks gene set enrichment analysis (GSEA) plots of IFNα and IFNγ pathways in 4-OHT–treated versus control samples. (**D**) Log_2_Fold change of selected cytokine genes from RNA-seq data in ARS-1620 (2 μM, 24 hours)–treated NCI-H358 cells versus dimethyl sulfoxide (DMSO) control. (**E**) MSigDB Hallmarks GSEA plots of IFNα and IFNγ pathways in ARS-1620–treated versus control samples. (**F**) Same analysis as (D) of RNA-seq from 3LL ΔNRAS cells treated with 100 nM MRTX1257 (24 hours, *n* = 3). (**G**) Same analysis as (E) of RNA-seq from 3LL ΔNRAS cells. All statistics represent false discovery rate (FDR)–adjusted *P* values (*q* < 0.05).

Previous reports have described that KRAS signaling can mediate the expression of cytokines such as IL-8 and GM-CSF in pancreatic cancer models (*22*). We therefore decided to assess the role of oncogenic KRAS signaling in the regulation of the expression of immunomodulatory factors in the lung. For this purpose, we made use of a cell line model of immortalized human lung pneumocytes expressing a tamoxifen-inducible oncogenic KRAS protein (KRAS^G12V-ER^) (fig. S1A) (*14*). Activation of oncogenic KRAS signaling induced the secretion of a number of cytokines and chemokines that could affect the recruitment and polarization of different immune cells (Fig. 1A). To further expand the scope of our investigation, we performed whole transcriptome analysis [RNA sequencing (RNA-seq)] on this model. Results validated the transcriptional induction of myeloid cell modulatory factors IL-8, CXCL1, CCL2, and GM-CSF and several other KRAS-regulated cytokines (Fig. 1B). In addition, gene set pathway analysis revealed a KRAS-dependent negative regulation of type I and type II IFN responses in lung pneumocytes (Fig. 1C and fig. S1B), previously shown to be crucial for antitumor immunity and sensitivity to immunotherapy (*23*, *24*), which could reflect a mechanism triggered by oncogenic KRAS to promote immune evasion.

We then assessed whether treatment with a therapeutic KRAS^G12C^ inhibitor could reverse these mechanisms. In two KRAS^G12C^-mutant human lung cancer cell lines, abrogation of oncogenic KRAS signaling by a KRAS^G12C^ inhibitor (fig. S1C) led to the down-regulation of cytokines and chemokines, particularly those involved in the recruitment and differentiation of myeloid cell populations, known to exert tumor-promoting effects in the TME (Fig. 1D and fig. S1D, top). Only the neutrophil chemoattractants CXCL2 and CXCL8 were consistently KRAS-regulated in both models, while most factors were cell line specific, suggesting that different cell lines exhibit different cytokine expression patterns. RNA-seq analysis of these cell lines also revealed that KRAS^G12C^ inhibition up-regulates IFNα and IFNγ response gene expression, a mechanism that was consistent across both cell lines (Fig. 1E and fig. S1D, bottom).

We decided to extend our findings to a murine cell line to use immunocompetent mouse models to examine the effects of oncogenic KRAS on antitumor immunity in vivo. We made use of a murine transplantable KRAS^G12C^-mutant lung cancer cell line derived from Lewis lung carcinoma, 3LL ΔNRAS [described in (*14*)], which is sensitive to KRAS^G12C^ inhibition (fig. S1E). Using this model, we validated the effect of KRAS^G12C^ inhibitors on the transcriptomic down-regulation of secreted immunomodulatory factors, by both RNA-seq (Fig. 1F) and quantitative polymerase chain reaction (qPCR; fig. S1F) analysis. Likewise, we validated the up-regulation of type I and II IFN gene sets observed in previous models (Fig. 1G and fig. S1G). Together, these data suggest that oncogenic KRAS signaling regulates the expression of factors that could affect the TME and antitumor immunity and highlight the role of KRAS^G12C^ inhibitors in reversing these potentially immune evasive mechanisms.

### KRAS signaling down-regulates IFN pathway gene expression via MYC

**Fig. 2.**
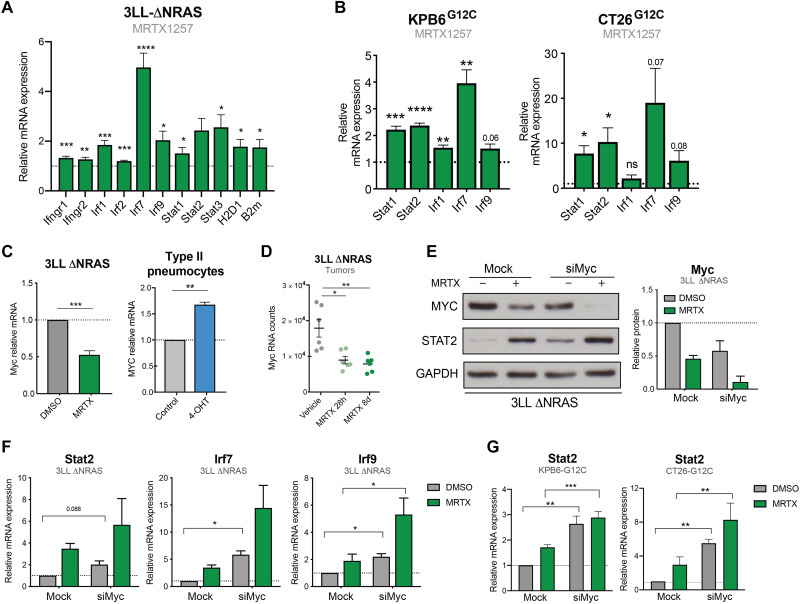
KRAS signaling down-regulates IFN pathway gene expression via MYC. (**A**) qPCR analysis of IFN-induced genes in MRTX1257-treated (100 nM, 24 hours) 3LL ΔNRAS cells (2^−ΔΔCT^, normalized to control sample for all genes, *n* = 6, unpaired *t* test, mean + SEM). (**B**) Same as (A) using KPB6^G12C^ (*n* = 4) and CT26^G12C^ (*n* = 3) cell lines. (**C**) qPCR showing KRAS-dependent regulation of Myc in 3LL ΔNRAS cells (*n* = 3) after treatment with MRTX1257 and KRAS^G12V-ER^ pneumocytes (*n* = 4) after treatment with 4-OHT (unpaired *t* test, mean + SEM). (**D**) RNA-seq mRNA counts of 3LL ΔNRAS lung tumors treated with vehicle or MRTX1257 (50 mg/kg) for 28 hours or 8 days (each dot represents a tumor, *n* = 6 per group, FDR *P* adjusted value). (**E**) Western blot showing MYC knockdown and STAT2 up-regulation of 3LL ΔNRAS cells treated with 100 nM MRTX1257 (24 hours), Myc small interfering RNA (siRNA; 48 hours), or both. Quantification for two independent experiments is shown on the right (mean + SEM). (**F**) qPCR analysis of IFN-induced genes in 3LL ΔNRAS cells treated with 100 nM MRTX1257, Myc siRNA, or both (2^−ΔΔCT^, normalized to control sample for all genes, *n* = 3, paired *t* tests, siMyc versus Mock, mean + SEM). (**G**) Same analysis as (F) in KPB6^G12C^ (*n* = 4) and CT26^G12C^ (*n* = 3) cells.

Next, we decided to further investigate the mechanistic link between KRAS signaling and IFN responses given their important role in antitumor immunity. We began by validating our RNA-seq finding that genes coding for components of the IFN response were up-regulated by KRAS^G12C^ inhibition in 3LL ΔNRAS cells (Fig. 2A). This up-regulation occurred in a MEK-dependent and cell viability–independent manner, beginning at approximately 6 hours after treatment and peaking at 24 hours after treatment (fig. S2, A to C). To extend our findings, we used two additional mouse cell lines modified to harbor KRAS^G12C^ mutations, the KRAS-mutant, p53-deleted lung cancer cell line KPB6^G12C^ (*25*) and the KRAS-mutant colon cancer cell line CT26^G12C^ (*19*). In these models, treatment with the KRAS^G12C^ inhibitor MRTX1257 (MRTX) consistently led to the up-regulation of canonical IFN signaling pathway genes (Fig. 2B). MEK inhibition in non-G12C mutant isogenic KPB6 cell lines also increased IFN signaling gene expression (fig. S2D), indicating that the mechanism is conserved across oncogenic KRAS mutations.

The conserved regulation of IFN signaling pathway genes after KRAS inhibition could suggest a direct cross-talk between oncogenic KRAS and IFN signaling pathways. IFNs bind their receptors on the membrane of target cells and drive transcriptional changes via activation of Janus kinase (JAK)–signal transducer and activator of transcription (STAT) signaling modules. To investigate whether the increase in gene expression in response to KRAS inhibition was a result of augmented IFN signaling, we examined the effect of blocking or depleting individual IFN pathway components. However, antibody-mediated blocking of the IFNα receptor (fig. S2E), pharmacological inhibition of JAK1/2 signal transduction with ruxolitinib (fig. S2F), and gene knockdown of Stat1 or Stat2 (fig. S2G) did not affect the MRTX-driven up-regulation of IFN genes (fig. S2H), suggesting that KRAS-driven inhibition of the IFN pathway occurs independently of the IFN receptors and JAK-STAT proteins.

A known negative transcriptional regulator of IFN genes is the MYC oncoprotein, which is also a RAS target (*26*). As expected, MYC mRNA levels were down-regulated by KRAS^G12C^ inhibition in vitro and in vivo and up-regulated after KRAS^G12V^ activation (Fig. 2, C and D), confirming the KRAS-driven regulation of MYC in our models. We assessed the role of MYC in the regulation of IFN signaling pathway genes by the KRAS^G12C^ inhibitor. In the 3LL ΔNRAS cell line, despite incomplete knockdown (Fig. 2E), MYC depletion was able to increase the expression of these genes (Fig. 2F). Because MRTX treatment led to a further down-regulation of MYC, increased gene expression was observed when MRTX and siMyc were combined. Up-regulation of IFN signaling pathway genes in response to MYC depletion was a common response observed across the three murine KRAS^G12C^ cell lines (fig. S2, I and J). Furthermore, in CT26^G12C^ and KPB6^G12C^ cells, where near-complete knockdown of MYC was achieved (fig. S2I), no significant additional effects on gene or protein expression were observed by combined siMyc and MRTX treatment (Fig. 2G), suggesting that MRTX-driven regulation of these genes is primarily through MYC. Together, these data suggest that KRAS^G12C^ inhibition, through down-regulation of MYC, leads to increased expression of genes associated with the IFN response.

### KRAS^G12C^ inhibition enhances tumor cell–intrinsic IFN responses

**Fig. 3.**
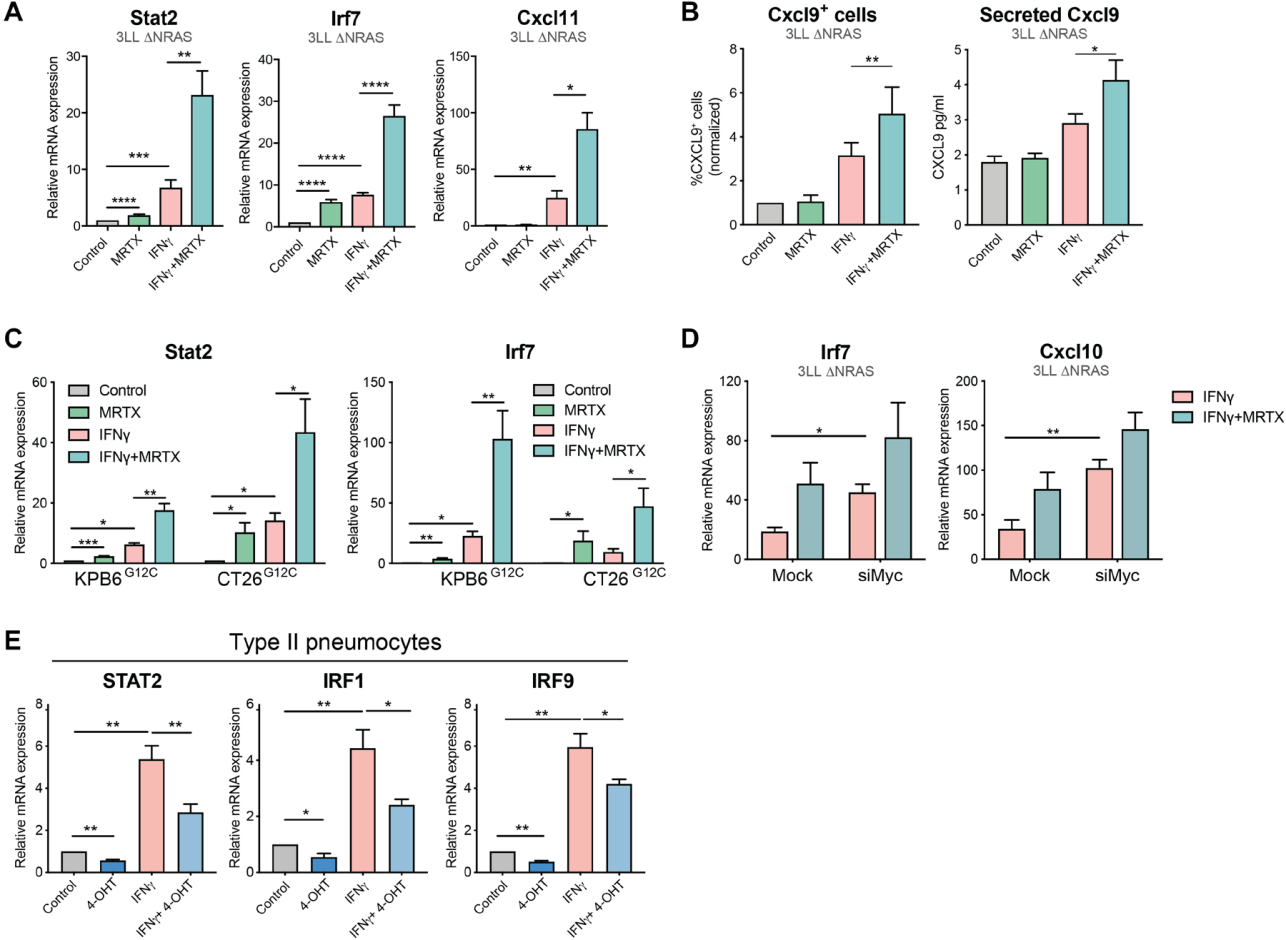
KRAS^G12C^ inhibition enhances tumor cell–intrinsic IFN responses. (**A**) qPCR analysis of IFN-induced genes in MRTX1257 (100 nM, 24 hours) and/or recombinant IFNγ (100 ng/ml)–treated 3LL ΔNRAS cells (2^−ΔΔCT^, normalized to control sample for all genes, *n* = 6, paired *t* test, mean + SEM). (**B**) Protein validation of IFN response regulation by KRAS. Left: Percentage of CXCL9-positive cells as measured by flow cytometry on 3LL ΔNRAS cells after treatment with MRTX1257 and/or IFNγ. Right: Concentration of CXCL9 secreted to the medium of 3LL ΔNRAS cells after treatment with MRTX1257 and/or IFNγ, measured by enzyme-linked immunosorbent assay (normalized to control sample, *n* = 3, paired *t* test, mean + SEM for both). (**C**) Same as (A) using KPB6^G12C^ (*n* = 4) and CT26^G12C^ (*n* = 3) cell lines. (**D**) qPCR analysis of IFN-induced genes in 3LL ΔNRAS cells treated with IFNγ only or IFNγ and MRTX1257 in the presence of 48 hours of Mock or Myc siRNA (2^−ΔΔCT^, normalized to IFNγ only–treated sample for all genes, *n* = 3, paired *t* test, mean + SEM). (**E**) qPCR analysis of IFN pathway genes in human KRAS^G12V-ER^ pneumocytes after treatment with 4-OHT and/or recombinant IFNγ for 24 hours (normalized to control sample, *n* = 4, paired *t* test, mean + SEM).

We next investigated whether KRAS^G12C^ inhibitor–driven changes in gene expression affected the capacity of tumor cells to respond to IFNγ. We found that IFNγ-driven transcriptional effects were enhanced by MRTX treatment (Fig. 3A). KRAS^G12C^ inhibition augmented the IFNγ-driven expression of immunomodulatory IFN-stimulated genes (ISGs) such as T cell chemoattractants *Cxcl9/10/11* and antigen presentation genes including *H2-d/k1*, *Ciita*, and *B2m* (Fig. 3A and fig. S3A). Consistent with the KRAS-dependent regulation of type I and II IFN responses observed in our RNA-seq analysis, MRTX treatment was likewise able to enhance IFNα- and IFNβ-driven gene expression (fig. S3B). These transcriptional changes also led to increased protein expression of ISG, as evidenced by an increased proportion of IFNγ-induced CXCL9-secreting tumor cells after treatment with MRTX (Fig. 3B). We validated that MRTX treatment enhanced IFNγ-driven gene (Fig. 3C) and protein (fig. S3, C and D) expression in two additional murine cell lines. A similar improvement of responses to IFNγ could be achieved by MYC knockdown (Fig. 3D), confirming the role of MYC in the regulation of IFN responses.

To exclude the possibility that reduced cell fitness contributed to the effects of KRAS^G12C^ inhibition on the response to IFNγ, we validated our findings in the KRAS^G12V-ER^ human pneumocyte cell line. In this model, we observed that 4-hydroxytamoxifen (4-OHT)–induced KRAS^G12V^ activation led to the down-regulation of IFN signaling pathway genes and was able to decrease IFNγ-driven transcriptional effects (Fig. 3E), suggesting a mechanistic link between the KRAS and IFN pathways, which is not influenced by cell viability.

In summary, we have shown that oncogenic KRAS signaling can suppress responses to IFN, and that this can be alleviated with pharmacological KRAS^G12C^ inhibition. KRAS inhibition in consequence leads to an increased sensitivity of tumor cells to type I and II IFNs, which translates to higher expression of IFN-induced genes such as T cell chemoattractants and antigen presentation genes that could positively affect antitumor immunity in vivo.

### KRAS^G12C^ inhibition in vivo remodels the highly immunosuppressive TME of 3LL ΔNRAS lung tumors

The results presented above demonstrate the ability of KRAS^G12C^ inhibitors to reverse KRAS-driven immune evasion mechanisms, such as enhancing tumor cell–intrinsic IFN responses and modulating the expression of secreted immunomodulatory factors. Next, we aimed to assess how inhibition of oncogenic KRAS in vivo can affect the composition of the TME in lung tumors.

**Fig. 4.**
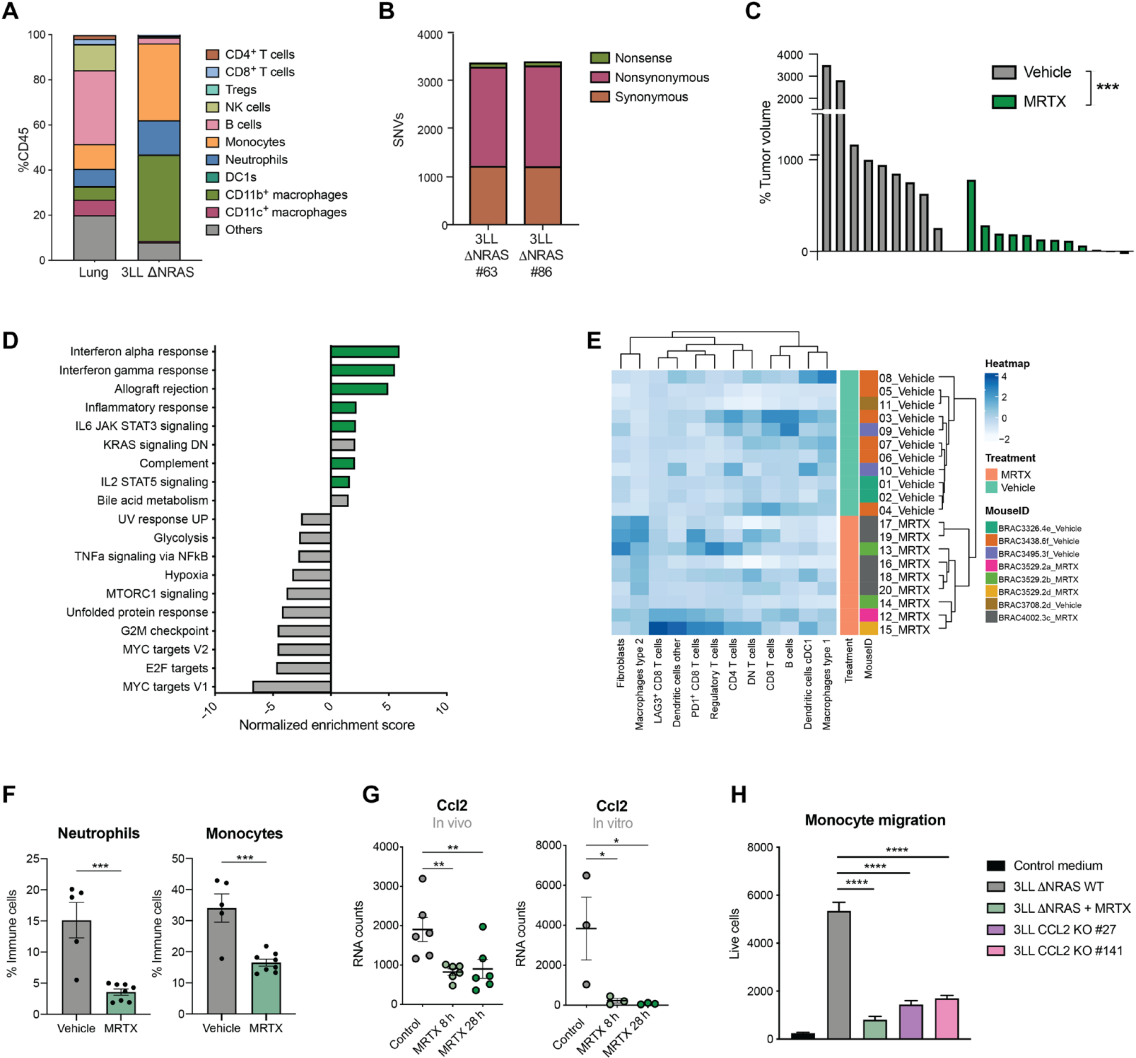
KRAS^G12C^ inhibition remodels the immunosuppressive TME of 3LL ΔNRAS lung tumors. (**A**) Immunophenotyping of dissected lung tumors obtained by intravenous administration of 3LL ΔNRAS cells (*n* = 5 mice) versus healthy lung tissue (*n* = 6 mice) obtained by flow cytometry. (**B**) Whole-exome sequencing SNV analysis of two NRAS CRISPR-edited 3LL clones. (**C**) Posttreatment tumor volume change as measured by μCT scanning of 3LL ΔNRAS lung tumors after 1 week of treatment with vehicle control or MRTX1257 (50 mg/kg) (each bar represents one tumor, Mann-Whitney test). (**D**) Summary of significantly (FDR *q* < 0.05) up- and down-regulated pathways in MRTX- versus vehicle-treated lung tumors (MSigDB Hallmarks). (**E**) Hierarchical clustering of relative frequencies of tumor-infiltrating cell types in MRTX- and vehicle-treated tumors obtained by IMC. (**F**) Percentage of neutrophils (gated as CD45^+^ CD11b^+^ Ly6C^+^ Ly6G^+^) and monocytes (gated as CD45^+^ CD11b^+^ Ly6C^hi^ Ly6G^−^) in vehicle-treated (*n* = 5) and MRTX-treated (*n* = 8) lung tumors measured by flow cytometry (each dot represents a mouse, unpaired *t* test). (**G**) mRNA counts for *Ccl2* gene in MRTX-treated 3LL ΔNRAS tumors (*n* = 6 per group, left) and cells (*n* = 3, right) obtained by RNA-seq (FDR-adjusted *P* value). (**H**) Live cell count (by flow cytometry) of bone marrow–derived monocytes that have migrated through a transwell in the presence of conditioned medium from 3LL ΔNRAS cells, MRTX-treated cells, or two clones from *Ccl2* CRISPR knockout [*n* = 3 independent experiments, one-way analysis of variance (ANOVA)].

The 3LL ΔNRAS cell line can form orthotopic tumors in the lungs of C57BL/6 mice when delivered intravenously. Immunophenotypic characterization of these tumors revealed a predominant infiltration of myeloid cells, known to exert immunosuppressive actions, while antitumorigenic cells like lymphocytes and natural killer (NK) were largely absent from the TME (Fig. 4A). Using IMC, we recently showed that there was an inclusion of macrophages and neutrophils in the core of 3LL ΔNRAS lung tumors, while effector cells remained at the tumor periphery (*21*). Consistent with this apparent immunosuppressive TME, growth of these tumors was not affected by a lack of B and T cells in Rag1^−/−^ mice (fig. S4A). Whole-exome sequencing of two 3LL ΔNRAS single-cell clones derived from the CRISPR-Cas9 deletion of NRAS (*14*) revealed that these cell lines harbor thousands of clonal somatic nonsynonymous single-nucleotide variations (SNVs) compared to the reference C57BL/6J genome (Fig. 4B). Whole-exome sequencing and RNA expression data were combined to perform in silico neoantigen prediction. Results showed that this cell line harbors numerous predicted neoepitopes with high or medium affinity for major histocompatibility complex (MHC) binding (fig. S4B), but flow cytometric analysis revealed that it has lost the expression of one of the MHC alleles, H2-Kb, while retaining the other, H2-Db (fig. S4C), possibly reflecting a mechanism to escape immunological rejection. It is noticeable that neoantigens predicted to bind to the absent H2-Kb are several-fold more highly represented than neoantigens predicted to bind to the expressed H2-Db. Therefore, we hypothesize that this cell line contains sufficient neoantigens to elicit an antitumor immune response, yet it is highly immune evasive and avoids rejection in immunocompetent hosts, likely through a combination of mechanisms including reduced neoantigen presentation and production of an immunosuppressive TME.

Treatment of 3LL ΔNRAS lung tumor–bearing mice with the KRAS^G12C^ inhibitor MRTX for 1 week resulted in marked tumor growth inhibition (Fig. 4C), although relatively few tumors actually decreased in size, highlighting the extreme aggressiveness of this tumor model. At this time point, we harvested tumors to perform RNA-seq analysis, flow cytometric analysis of immune cell infiltration, and IMC to examine the effects of KRAS^G12C^ inhibition on the TME of this highly immunosuppressive model. As anticipated from the in vitro data, gene set enrichment analysis of 3LL ΔNRAS lung tumors treated with MRTX revealed an up-regulation of several immune-related pathways, including IFNα and IFNγ responses, IL-2 and IL-6 signaling, allograft rejection, and complement and inflammatory responses (Fig. 4D). We were likewise able to confirm the KRAS-dependent regulation of IFN signaling pathway gene sets in vivo (fig. S4D). This marked remodeling of the TME was confirmed by IMC analysis, where hierarchical clustering based on immune cell infiltration patterns was able to discern vehicle- and MRTX-treated samples (Fig. 4E).

KRAS^G12C^ inhibition was able to significantly reduce the high infiltration of myeloid cells like monocytes and neutrophils observed in this lung tumor model, as measured by flow cytometry (Fig. 4F). We then wondered whether the down-regulation of tumor cell–intrinsic cytokine expression observed in vitro (Fig. 1F) could play a role in the regulation of myeloid cell infiltration. The strongest KRAS-regulated cytokine in the 3LL ΔNRAS cells in vitro was *Ccl2*, a canonical chemoattractant for monocytes. The KRAS-dependent regulation of CCL2 secretion was also validated in our KRAS^G12V-ER^ pneumocyte cell line (fig. S4E). Furthermore, in vivo MRTX treatment of 3LL ΔNRAS tumor–bearing mice led to a significant down-regulation of *Ccl2* expression in the tumor (Fig. 4G), suggesting that tumor cells may be one of the main sources of this cytokine in the TME. To validate the role of KRAS-mediated regulation of CCL2 in the changes observed in the TME, we measured monocyte migration ex vivo. Results showed that migration was significantly abrogated when bone marrow–derived monocytes were cultured in conditioned medium from MRTX-treated cells and to a similar extent when cultured in medium from *Ccl2*^−/−^ cells (Fig. 4H). These data suggest that tumor cell–specific KRAS^G12C^ inhibition, via inhibition of the secretion of CCL2, leads to an impaired recruitment of monocytes into the TME, which could constitute a mechanism that alleviates immunosuppression.

### KRAS^G12C^ inhibition in vivo increases T cell infiltration and activation

**Fig. 5.**
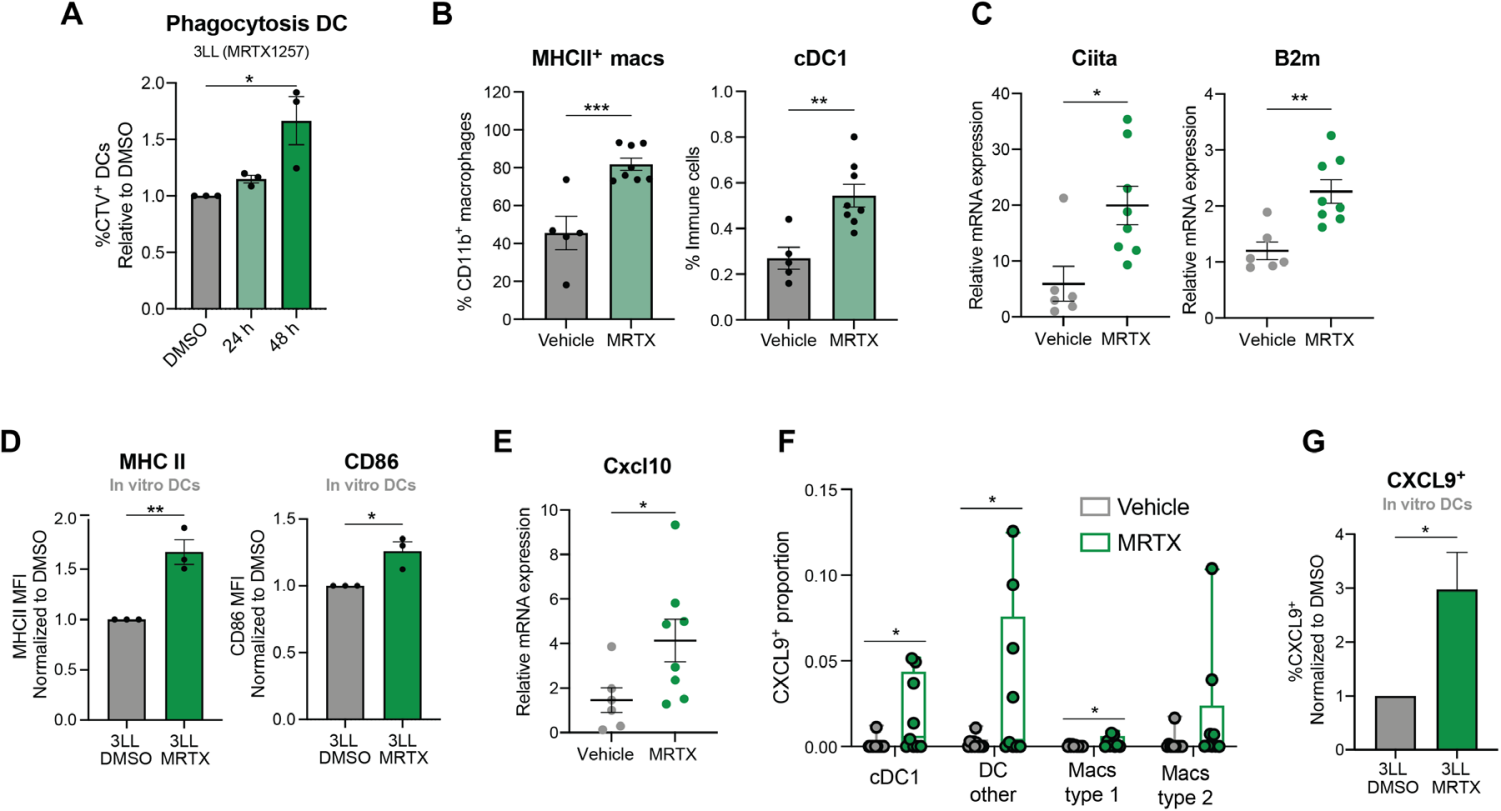
KRAS^G12C^ inhibition promotes APC activation. (**A**) Normalized percentage of GFP^+^ Mutu DCs that have phagocytosed CTV^+^ 3LL ΔNRAS cells, previously treated with DMSO control, or 100 nM MRTX1257 for 24 or 48 hours, measured by flow cytometry (*n* = 3 independent experiments, one-way ANOVA, mean ± SEM). (**B**) Flow cytometry analysis of 3LL ΔNRAS lung tumors treated with vehicle or MRTX1257 (50 mg/kg) for 7 days. Macrophages are gated as Live CD45^+^ CD11b^+^ CD24^−^ CD64^+^, and cDC1s are obtained by Live, CD45^+^, CD11c^+^ CD24^+^ CD103^+^ gating (*n* = 5 for vehicle, *n* = 8 mice for MRTX-treated, unpaired *t* test, mean ± SEM). (**C**) qPCR data for 3LL ΔNRAS lung tumors treated as in (B) (2^−ΔΔCT^, unpaired *t* test, *n* = 7 vehicle, *n* = 8 treated, mean ± SEM). (**D**) Normalized mean fluorescence intensity of MHCII and CD86 as measured by flow cytometry of DCs cocultured with 3LL ΔNRAS cells previously treated with either DMSO or MRTX for 48 hours (pregated as GFP^+^, unpaired *t* test, *n* = 3 independent experiments, mean ± SEM). (**E**) qPCR data of Cxcl10 gene in 3LL ΔNRAS tumors, analyzed as in (C). (**F**) Proportion of CXCL9^+^ cells in each population, as detected by IMC, per ROI (Region of Interest) (*n* = 11 vehicle, *n* = 9 MRTX, unpaired *t* tests, mean ± SEM). (**G**) Normalized percentage of CXCL9^+^ DCs after coculture with 3LL ΔNRAS cells as in (D) (*n* = 5 independent experiments, unpaired *t* test, mean + SEM).

While a reduction in immunosuppressive populations in the TME constitutes a mechanism to improve antitumor immunity, immunological rejection can only be achieved by the specific activation of cytotoxic populations such as CD8^+^ T cells. For the generation of an adaptive immune response, lymphocytes need to be primed by professional APCs, which, in turn, need to have been activated themselves by engulfment of tumor-specific antigens. Therefore, we sought to assess whether the reduction of viability and increase of apoptosis caused by KRAS^G12C^ inhibition [(*14*) and fig. S1E] could affect dendritic cell (DC) activity in vitro. The green fluorescent protein–positive (GFP^+^) Mutu DCs were able to phagocytose MRTX-treated CellTrace Violet (CTV)–labeled 3LL ΔNRAS cells when cocultured (Fig. 5A), which constitutes the first step of DC activation. In addition, we found that in vivo MRTX treatment increased the presence of APCs in the TME (Fig. 5B), with a consistent increase in the expression of genes involved in antigen presentation (Fig. 5C and fig. S5A). Consistent with this finding, coculture of tumor cells with DCs revealed that KRAS^G12C^ inhibition promoted the up-regulation of activation markers MHCII and CD86 on DCs (Fig. 5D). MHC II up-regulation seemed to be mediated by tumor cell–secreted factors, while CD86 required the presence of tumor cells, suggesting that different mechanisms might be at play in the tumor cell–mediated activation of DCs (fig. S5B).

Activated DCs are known to secrete CXCR3 ligands (CXCL9/10/11), a prominent feature of inflamed TMEs (*27*). We found that KRAS^G12C^ inhibition in vivo resulted in a higher expression of these T cell chemoattractants and their receptor (Fig. 5E and fig. S5C). IMC analysis in the 3LL ΔNRAS lung tumors revealed that CXCL9 was mainly expressed in cells that also expressed markers for APCs (fig. S5D). Furthermore, while these tumors had negligible basal CXCL9 expression, CXCL9-expressing cells were mostly found among DCs and macrophages in a subset of the MRTX-treated tumors (Fig. 5F). Mechanistically, we observed that the coculture with MRTX-treated tumor cells was able to lead to the up-regulation of CXCL9 in DCs in vitro (Fig. 5G). This could not be recapitulated by conditioned medium incubation, suggesting that signals from MRTX-treated tumor cells, other than secreted factors, mediate this up-regulation (fig. S5E).

**Fig. 6.**
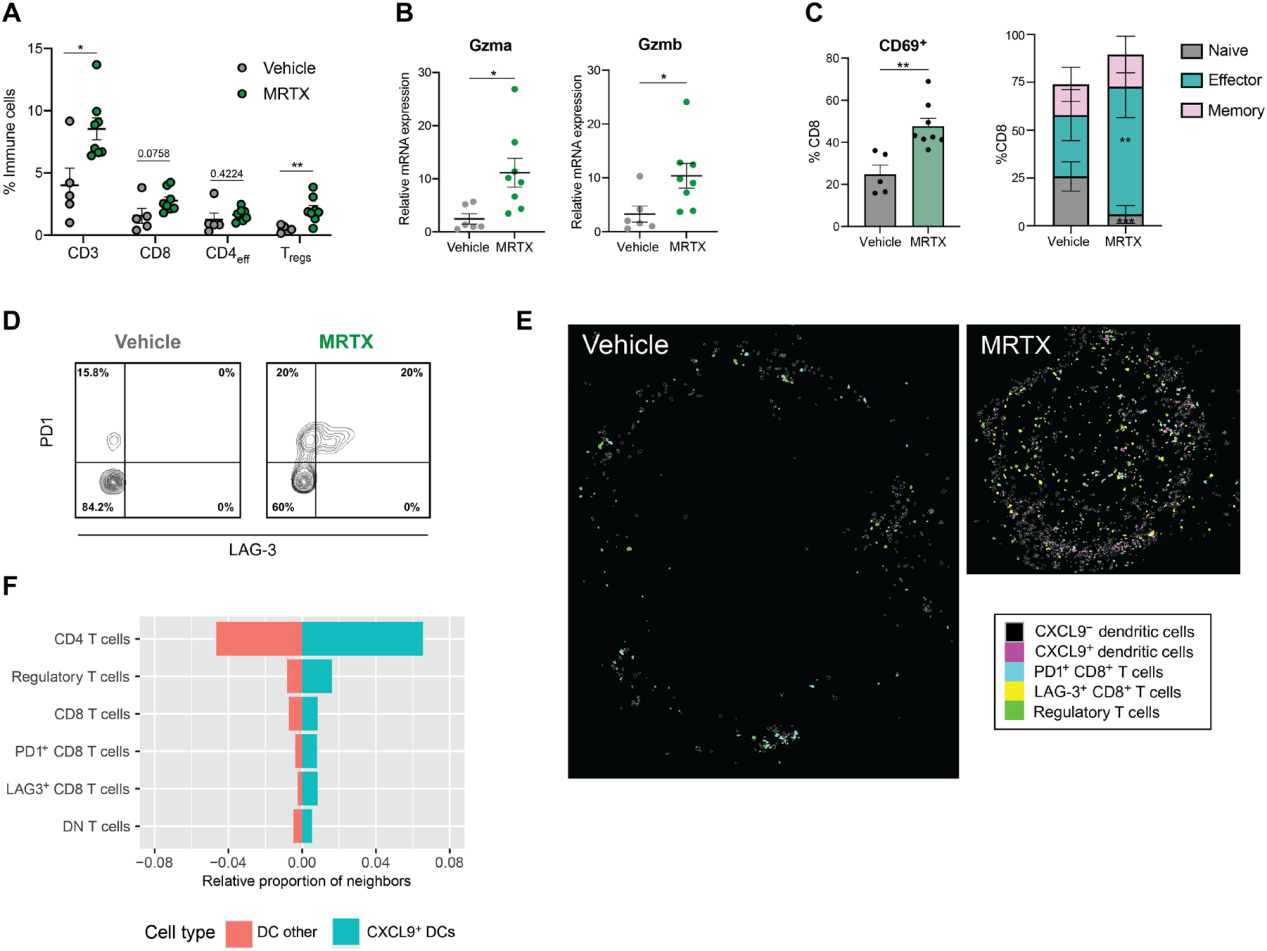
KRAS^G12C^ inhibition leads to T cell infiltration and activation. (**A**) Summary of T cell infiltration measured by flow cytometry in vehicle versus MRTX-treated lung tumors (*n* = 5 for vehicle, *n* = 8 mice for treated, unpaired *t* tests). (**B**) qPCR analysis of cytotoxicity genes in 3LL ΔNRAS lung tumor (2^−ΔΔCT^, unpaired *t* test, *n* = 7 vehicle, *n* = 8 treated, mean ± SEM). (**C**) Flow cytometry analysis of CD8^+^ T cell phenotypes. Left: Percentage of CD69^+^ CD8^+^ T cells in both treatment groups (*n* = 5 vehicle, *n* = 8 MRTX-treated, unpaired *t* test, mean ± SEM). Right: Percentage of naïve (CD44^−^ CD62L^+^), effector (CD44^+^ CD62L^−^), and memory (CD44^+^ CD62L^+^) CD8^+^ T cells, same analysis as on the left for each cell population. (**D**) Contour plot of PD1 and LAG-3 expression on CD8^+^ cells in vehicle- and MRTX-treated 3LL ΔNRAS lung tumor samples (graph shows one representative example for *n* = 5 vehicle and *n* = 8 MRTX-treated samples). (**E**) Visualization of cell outlines as measured by IMC, of CXCL9-negative and CXCL9-positive DCs, PD1^+^ and LAG-3^+^ CD8^+^ T cells, and T_regs_ in a vehicle- and MRTX-treated tumor. (**F**) Quantification of occurrence of the different T cell subsets in the neighborhood of CXCL9^+^ and CXCL9^−^ DCs, depicted as the average proportion of that cell type among all neighbors within 100-pixel radius of the DC subset.

Next, we evaluated whether the observed activation of APCs coincided with changes in the T cell compartment. We observed that the presence of all T cell compartments, particularly Foxp3^+^ regulatory T cells (T_regs_), was increased by MRTX in these lung tumors (Fig. 6A). Consistent with the increased T cells, cytotoxicity genes were also significantly up-regulated in MRTX-treated tumors (Fig. 6B). NK cell infiltration was also increased in treated tumors (fig. S5F), which could be contributing to the increased expression of cytotoxicity genes. This increase in cytotoxicity was confirmed by the significantly increased presence of CD69^+^ and antigen-experienced (effector and memory) CD8^+^ T cells observed after MRTX treatment (Fig. 6C and fig. S5G).

Previous reports have shown that KRAS inhibition triggers an improved immune response that drives T cell exhaustion, resulting in sensitivity to ICB in immunogenic models of KRAS^G12C^-mutant cancer (*8*, *19*). In our immune evasive 3LL ΔNRAS lung tumor model, we also observed via flow cytometry that PD1^+^ T cells were significantly increased after MRTX treatment (fig. S5H). A subset of these cells also expressed LAG-3 (Fig. 6D), and we likewise found an up-regulation of several other T cell exhaustion genes in our RNA-seq analysis (fig. S5I).

As CXCL9 expression by DCs was previously described to be crucial to attract effector T cells (*27*), we further explored the relationship between the CXCL9^+^ DCs and the presence of different T cell subsets in the MRTX-treated tumors. There was a clear correlation between the abundance of CXCL9^+^ DCs and CD8^+^ T cells expressing PD1 and LAG-3 as well as T_regs_ (fig. S5J). Using the spatial information captured by IMC, we could also see that these cells are regularly found in close proximity to each other, with a clear enrichment of T_regs_ and CD8^+^ T cells with an exhausted phenotype in the direct neighborhood of CXCL9^+^ DCs compared to CXCL9^−^ DCs (Fig. 6, E and F). Whether the CXCL9-expressing DCs play a role in recruiting these effector cells, or that activated T cells locally produce IFNγ that in turn induces the CXCL9 expression in the DCs, cannot be deduced from these data.

Together, these data show that tumor cell–specific KRAS^G12C^ inhibition in a mouse lung cancer model leads to a more inflamed TME, evidenced by an activation of APCs and a strong increase in the presence of activated T cells that could not only exert cytotoxic actions on the tumor cells but also display an exhausted phenotype.

### KRAS^G12C^ inhibition synergizes with checkpoint blockade only in intrinsically immunogenic tumors

**Fig. 7.**
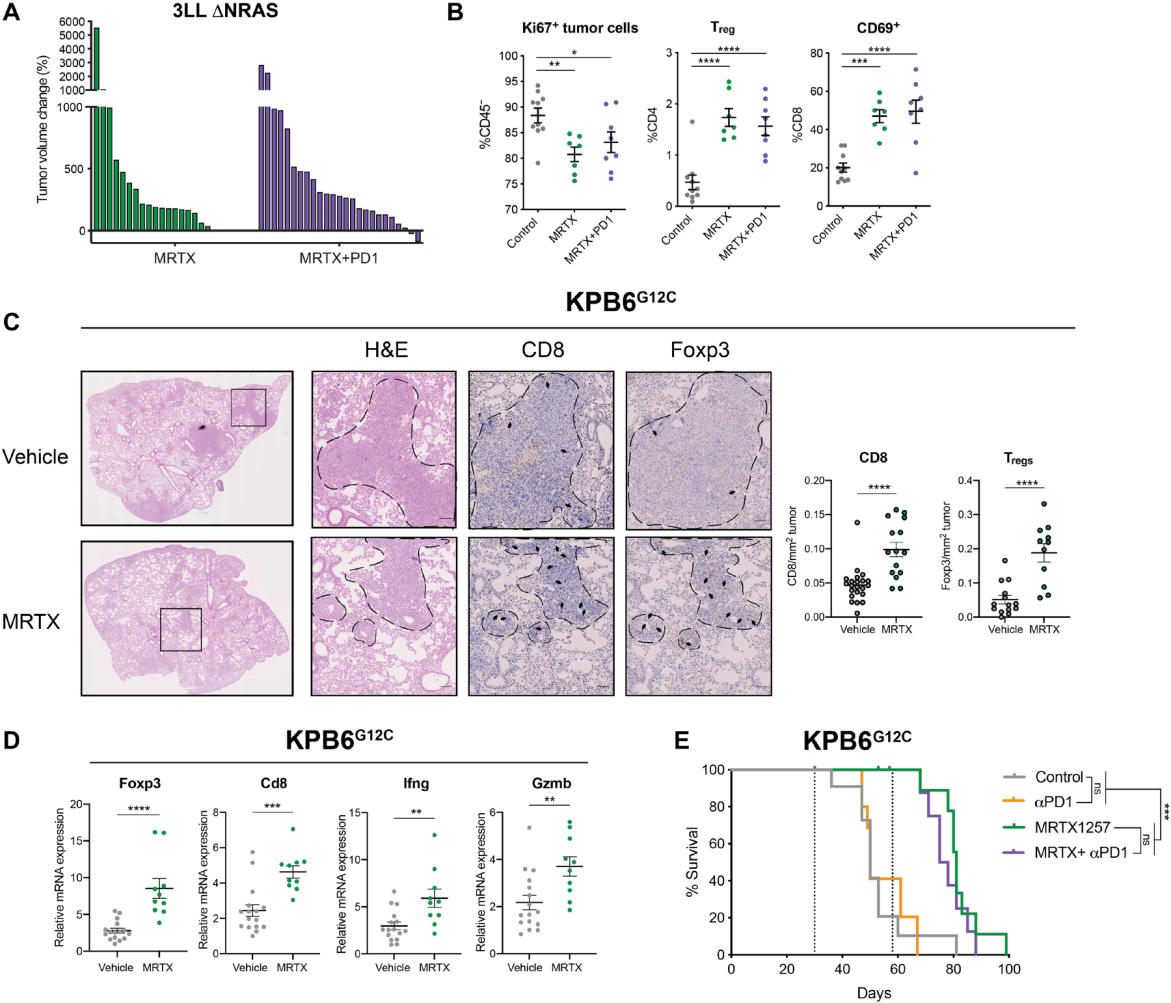
KRAS^G12C^ inhibition does not synergize with ICB in immune refractory tumors. (**A**) Tumor volume change after 2 weeks of treatment of 3LL ΔNRAS tumor–bearing mice with either MRTX1257 (50 mg/kg) only (*n* = 9 mice) or MRTX1257 and anti-PD1 (*n* = 10 mice). Each bar represents a tumor. (**B**) Flow cytometry analysis of proliferating tumor cells (CD45^−^ Ki67^+^), T_regs_ (CD3^+^ CD4^+^ Foxp3^+^), and activated T cells (CD69^+^ CD8^+^) in vehicle (*n* = 10)–treated, MRTX (*n* = 7)–treated, or MRTX plus anti-PD1 (*n* = 8)–treated (2-week treatment) 3LL ΔNRAS lung tumors (one-way ANOVA, mean ± SEM). (**C**) Immunohistochemistry analysis and quantification for CD8 (*n* = 4 mice per group) and Foxp3 (*n* = 3 mice per group) in KPB6^G12C^ tumor–bearing lungs after 7 days of vehicle or MRTX1257 treatment (50 mg/kg; each dot represents one tumor, unpaired *t* test, mean ± SEM). (**D**) qPCR analysis of immune genes in vehicle (*n* = 15 tumors) or MRTX-treated (*n* = 10 tumors) KPB6^G12C^ lung tumors (2^−ΔΔCT^, unpaired *t* test, mean ± SEM). (**E**) Survival of KPB6^G12C^lung tumor–bearing mice treated with vehicle [+immunoglobulin G (IgG) control, *n* = 6 mice], MRTX1257 (+IgG control, *n* = 6 mice), anti-PD1 (*n* = 5 mice), or combination (*n* = 4 mice, log-rank Mantel Cox test).

An increased presence of exhausted T cells and augmented IFN responses suggest that MRTX treatment has the potential to sensitize these tumors to ICB. Nevertheless, in this immune-resistant model (*28*), addition of an anti-PD1 antibody (Fig. 7A) or a combination of anti–PD-L1 and anti–LAG-3 antibodies (fig. S6A) did not improve the response to KRAS^G12C^ inhibition alone, nor did it enhance the TME remodeling driven by KRAS^G12C^ inhibition (Fig. 7B). We found that the lack of therapeutic response observed was not due to insufficient antigen presentation by the tumor cells, as reexpression of the epigenetically silenced H2-Kb by treatment with the DNA methyltransferase inhibitor decitabine in these cells did not improve responses to MRTX + PD1 (fig. S6, B and C).

To extend our findings, we made use of the KPB6^G12C^ cell line, which has been established from the KRAS^LSL_G12D/+^;Trp53^fl/fl^ mice (KP) and genetically engineered to express a KRAS^G12C^ mutation (*25*). Because of the very low number of clonal somatic SNVs, this model develops immune cold lung tumors (*25*). Orthotopic KPB6^G12C^ lung tumors were highly sensitive to KRAS^G12C^ inhibition (fig. S6D). Treatment of KPB6^G12C^ lung tumor–bearing mice with MRTX for a week led to increased T cell infiltration into the tumors (Fig. 7C) accompanied by an up-regulation of immune genes (Fig. 7D). Similar to our findings in 3LL ΔNRAS, we found a significant increase in CD8^+^ T cell and T_regs_ and increased *Ifng* and *Gzmb* expression, suggesting increased cytotoxicity. However, in this alternative immune resistant model, no synergism occurred between KRAS^G12C^ inhibition and ICB, and mice in all treatment groups succumbed to disease (Fig. 7E). We therefore concluded that despite the profound TME remodeling triggered by KRAS^G12C^ inhibition, it may not be sufficient to render highly immune resistant tumors sensitive to ICB.

**Fig. 8.**
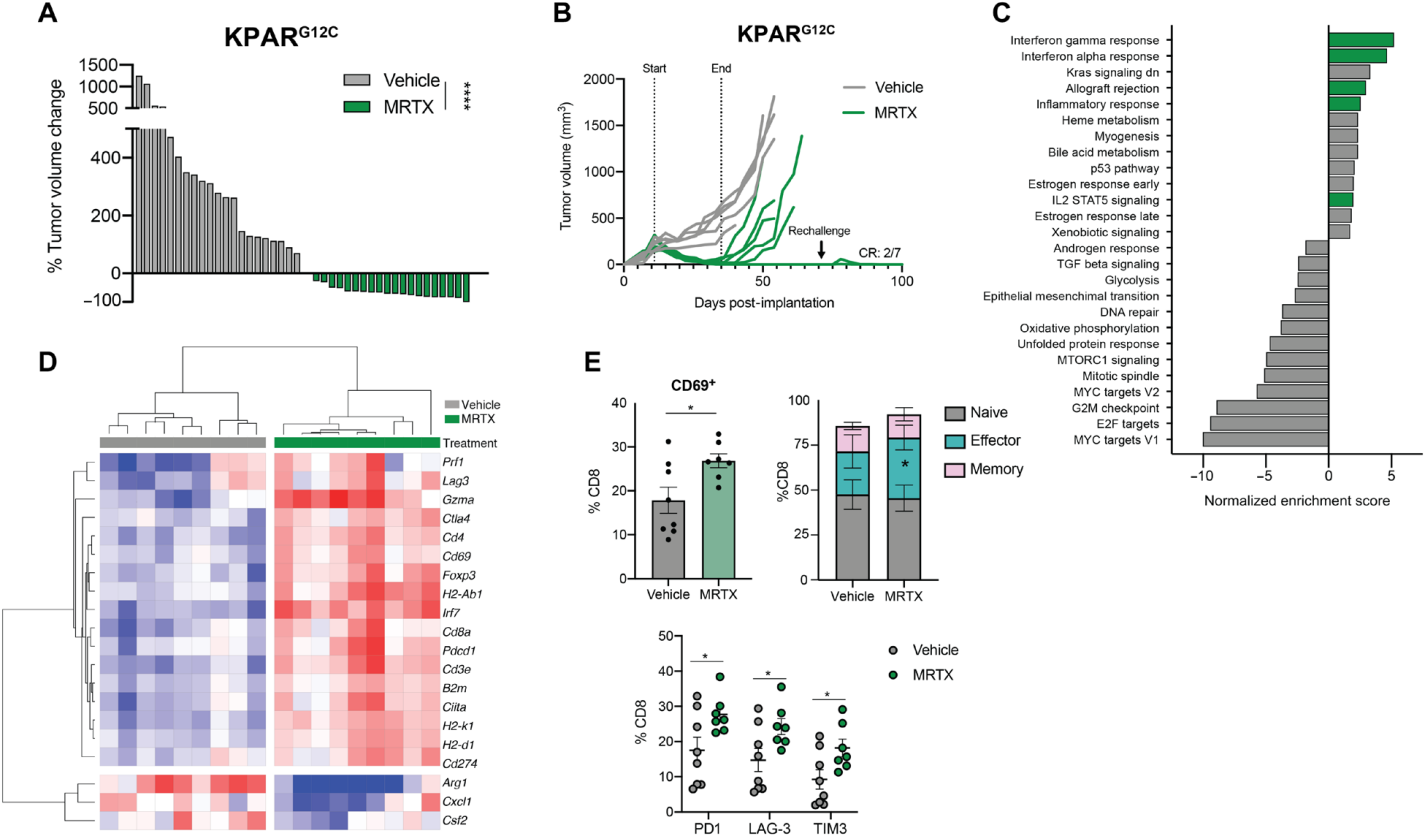
In an immunogenic model, MRTX-driven immune responses drive complete tumor rejection. (**A**) Tumor volume change after 7 days of treatment of KPAR^G12C^ tumor–bearing mice with either vehicle (*n* = 3 mice) or MRTX849 (*n* = 2 mice). Each bar represents a tumor, Mann-Whitney test. (**B**) Growth of subcutaneously implanted KPAR^G12C^ tumors treated with either vehicle or MRTX849 (50 mg/kg) for 2 weeks. At day 71, the remaining mice were rechallenged with KPAR^G12C^ cells in the opposite flank, which did not give rise to tumors. (**C**) Summary of significantly (FDR *q* < 0.05) up- and down-regulated pathways in MRTX849 (50 mg/kg, 6 days) versus vehicle-treated KPAR^G12C^ lung tumors (MSigDB Hallmarks); *n* = 9 tumors per group (three mice). (**D**) Heatmap showing mRNA expression from RNA-seq of KPAR^G12C^ tumors treated for 6 days with MRTX849 (50 mg/kg). Gene expression is scaled across all tumors. (**E**) Flow cytometry analysis of KPAR^G12C^-bearing lungs treated with either vehicle (*n* = 8 mice) or MRTX849 (50 mg/kg; *n* = 7 mice) for 6 days, showing increased CD69^+^ CD8^+^ T cells (top left), increased CD44^+^ CD62L^−^ effector CD8^+^ T cells (top right), and increased checkpoint molecule expression on CD8^+^ T cells (below) after KRAS inhibition (all statistics are Student’s *t* tests, mean ± SEM).

We therefore investigated the effects of KRAS^G12C^ inhibition in a new immunogenic model of KRAS-mutant lung cancer. The KPAR^G12C^ cell line has been shown to be immunogenic as its growth is impaired by the adaptive immune system (*25*). While sensitivity to KRAS^G12C^ inhibition in vitro was reduced compared to the 3LL ΔNRAS cell line (fig. S7A), the responses seen in vivo were much stronger, with most lung tumors shrinking more than 75% (Fig. 8A). Treatment of subcutaneous KPAR^G12C^ tumor–bearing mice with a KRAS^G12C^ inhibitor also resulted in outstanding tumor control, with two of seven mice achieving complete responses (Fig. 8B). These responders were resistant to tumor rechallenge, suggesting the development of immune memory. In contrast, the responses of 3LL ΔNRAS tumor–bearing mice to KRAS^G12C^ inhibition were not supported by the adaptive immune system. There were no long-term responses, with all mice relapsing on treatment, and responses were comparable in immunocompetent and immunodeficient mice (fig. S7B).

We then assessed the effects of KRAS^G12C^ inhibition on the remodeling of the TME in KPAR^G12C^ lung tumors. RNA-seq from MRTX-treated tumors showed an up-regulation of immune-related gene sets, confirming our observations in the 3LL ΔNRAS and KPB6^G12C^ models (Fig. 8C). Genes encoding for T cell infiltration (*Cd3e*, *Cd4*, *Cd8a*, and *Foxp3*), T cell activation (*Prf1*, *Cd69*, *Gzma*, *Pdcd1*, *Ctla4*, and *Lag3*), IFN responses (*Irf7*, *Irf9*, and *Cd274*), and antigen presentation (*H2-Ab1*, *H2-K1*, *H2-D1*, *Ciita*, and *B2m*) were up-regulated after treatment, while immunosuppressive cytokines (*Cxcl1* and *Csf2*) and markers of tumor-promoting myeloid populations (*Arg1*) were down-regulated (Fig. 8D and fig. S7C). Flow cytometric analysis revealed a significant up-regulation of activated, antigen-experienced, and exhausted T cells (Fig. 8E) and NK cells (fig. S7D) as well as a remodeling of the myeloid compartment, with a reduction of neutrophils and increased APC activation (fig. S7D) similar to previous models examined.

**Fig. 9.**
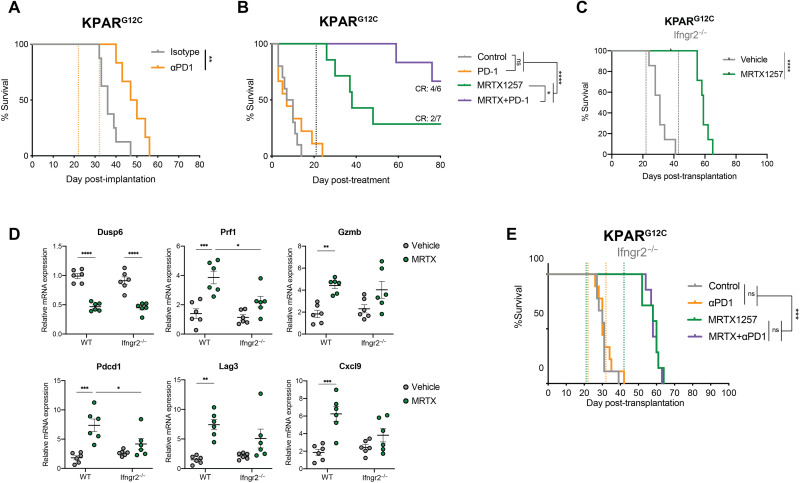
Synergy with anti-PD1 requires an intact tumor cell–intrinsic IFN response. (**A**) Survival of KPAR^G12C^ lung tumor–bearing mice after treatment with IgG control (*n* = 8 mice) or anti-PD1 (10 mg/kg; *n* = 6 mice). Dotted lines represent start and end of treatment, respectively, log-rank Mantel Cox test. (**B**) Survival of KPAR^G12C^ lung tumor–bearing mice after treatment with vehicle (+IgG control, *n* = 6 mice), anti-PD1 (10 mg/kg; *n* = 8 mice), MRTX1257 (50 mg/kg; +IgG control, *n* = 4 mice), or both (*n* = 6 mice). Dotted line represents end of treatment, log-rank Mantel Cox test. (**C**) Survival of KPAR^G12C^ *Ifngr2*^−/−^ lung tumor–bearing mice after treatment with vehicle or MRTX1257 (50 mg/kg; *n* = 7 mice per group). Dotted lines represent treatment start and end, respectively, log-rank Mantel Cox test. (**D**) qPCR analysis of KPAR^G12C^ WT or *Ifngr2*^−/−^ lung tumors treated with vehicle or MRTX1257 for 4 days (*n* = 6 tumors per group, mean ± SEM, 2^−ΔΔCt^). Each dot represents a lung tumor, one-way ANOVA. (**E**) Survival of KPAR^G12C^ Ifngr2^−/−^ lung tumor–bearing mice after treatment with vehicle (+IgG control, *n* = 9 mice), anti-PD1 (10 mg/kg; *n* = 9 mice), MRTX1257 (50 mg/kg; +IgG control, *n* = 7 mice), or both (*n* = 7 mice). Dotted line represents start and end of treatment for MRTX (green) and anti-PD1 (orange), log-rank Mantel Cox test.

In this immunogenic model, where early treatment with anti-PD1 alone confers therapeutic benefit (Fig. 9A), treatment of orthotopic tumor–bearing mice with MRTX alone led to complete responses in 28% of the mice (Fig. 9B). Furthermore, the percentage of complete responders was improved (66%) when KRAS^G12C^ inhibition was administered together with anti-PD1 immunotherapy treatment, even while treatment started later at a time point when single anti-PD1 therapy was no longer effective (Fig. 9B). The synergy between KRAS^G12C^ inhibition and anti-PD1 in this model was also reflected by the composition of the TME, with a further increase in immune infiltration and activation genes observed in the combination treatment (fig. S7E).

Using the KPAR^G12C^ cell line, we validated the KRAS-dependent regulation of IFN responses, driven through MYC (S7F), highlighting the universality of this mechanism. We then made use of the immunogenicity of this model and examined the role of tumor cell–intrinsic IFNγ signaling in the long-term therapeutic effect of KRAS^G12C^ inhibitors. To this end, we generated *Ifngr2*^−/−^ KPAR^G12C^ cells (fig. S7G), which are insensitive to IFNγ, while the KRAS inhibitor–driven up-regulation of IFN genes remains unaffected (fig. S7, H and I). We observed that complete responses to KRAS^G12C^ inhibition in vivo were dependent on tumor cell–intrinsic IFN signaling, as all mice bearing tumors formed by *Ifngr2*^−/−^ KPAR^G12C^ cells relapsed after MRTX treatment (Fig. 9C), while their sensitivity to KRAS inhibition in vitro remained unaffected (fig. S7J). Similarly, KRAS target gene *Dusp6* reduction in vivo was comparable in KPAR^G12C^ wild-type (WT) and *Ifngr2*^−/−^ tumors (Fig. 9D, top left). On the contrary, we observed that in *Ifngr2*^−/−^ tumors, the increase in T cell cytotoxicity (*Prf1* and *Gzmb*), activation (*Pdcd1* and *Lag3*), and myeloid cell activation (*Cxcl9*) in response to KRAS^G12C^ inhibition was significantly attenuated, probably contributing to the decreased long-term therapeutic efficacy of the inhibitor in this model. Furthermore, the synergism between MRTX and anti-PD1 treatment observed in this model was completely abrogated in *Ifngr2^−/−^* tumors (Fig. 9E).

Together, these data suggest that in an immunogenic tumor, KRAS^G12C^ inhibition can stimulate antitumor immunity, drive complete tumor rejection in a subset of mice, and sensitize tumors to ICB, resulting in increased complete responses when both treatments are combined. In addition, this process is dependent on the tumor cell–intrinsic ability to respond to IFNγ, which is regulated by KRAS signaling and contributes to long-term therapeutic efficacy of KRAS^G12C^ inhibition.

## DISCUSSION

KRAS^G12C^ inhibitors have shown promising clinical activity in KRAS^G12C^-mutant NSCLC patients (*9*, *10*). However, similarly to other targeted therapies, early clinical results indicate that drug resistance frequently arises, resulting in clinical relapses (*11*, *12*). KRAS^G12C^ inhibitors not only affect the survival of cancer cells but also can mediate immunomodulatory effects by reversing KRAS-driven immunosuppressive mechanisms and generate a TME that is more favorable for an antitumor immune response (*8*, *19*, *21*). This knowledge has served as a rationale to investigate clinical combinations of KRAS^G12C^ inhibitors with anti-PD1 or PD-L1 antibodies (*29*). However, previous studies have only validated this combination using mouse models that are highly ICB responsive (*8*, *19*). Here, we show that despite the profound TME remodeling caused by KRAS^G12C^ inhibition, this drug combination may not be sufficient to elicit durable responses in tumor models that are intrinsically resistant to ICB.

Understanding the mechanism of action of KRAS^G12C^ inhibitors and how they can modulate the TME may lead to the identification of additional combination strategies for those patients who will not benefit from the dual inhibition of KRAS^G12C^ and PD1. Our analysis has gained insight into the different mechanisms by which oncogenic KRAS signaling mediates immune evasion in lung cancer. It has already previously been described that oncogenic KRAS regulates the expression of cytokines and chemokines that can modulate the TME (*22*, *30*, *31*). Here, we show that KRAS^G12C^ inhibition reduces the secretion of monocyte and neutrophil chemoattractants by the tumor cells, which results in an impaired infiltration of these immunosuppressive cell types in the TME. Reprogramming myeloid populations by targeting selected cytokines or their receptors, like CCR2 (*32*) or CXCR2 (*33*), has been proposed as a mechanism to enhance response to immunotherapies (*34*). Treatment with KRAS^G12C^ inhibitors leads to modulation of various C(X)CL ligands secreted by tumor cells and can thus indirectly reduce immunosuppressive populations without associated toxicities. However, the identity of the KRAS-regulated cytokines appears to vary between tumor types.

Another mechanism by which oncogenic KRAS drives immune evasion is by inhibiting IFN responses. We have shown that KRAS^G12C^ inhibitor treatment releases the inhibition of IFN signaling pathway genes in all the models that we have analyzed. Moreover, activation of oncogenic KRAS in type II pneumocytes inhibits IFN pathway expression, suggesting that this is a conserved mechanism in the lung. Mechanistically, KRAS inhibits IFN gene expression via regulation of the oncogene MYC, which is consistent with previous observations in pancreatic cancer (*26*). KRAS^G12C^ inhibition enhances tumor cell sensitivity to type I and II IFNs and results in an increased IFN pathway activation in vivo. This is especially important as IFN responses are crucial for antitumor immunity and clinical responses to immunotherapies (*23*, *24*, *35*, *36*). By knocking out the IFNγ receptor in tumor cells, we have demonstrated that tumor cell–intrinsic IFN signaling is necessary to achieve long-lasting therapeutic responses to KRAS^G12C^ inhibitors in vivo. We have therefore expanded beyond the known role of IFN signaling in the response to immune therapies (*37*, *38*), showing that an intact IFN response is also required for durable immune responses to a targeted therapy such as KRAS^G12C^ inhibition.

As a consequence of the KRAS-dependent regulation of IFN responses, treatment with KRAS^G12C^ inhibitors increases antigen presentation. Oncogenic KRAS has previously been linked to reduced expression of MHC class I molecules (*39*, *40*). Reversion of this immune evasion mechanism can boost T cell recognition, rendering tumor cells more susceptible to immune cell attack. In addition, the cell death induced by KRAS^G12C^ inhibitors could also trigger an adaptive T cell response because of the release of dead cell–associated antigens. Consistent with this, we observe both in vitro and in vivo that KRAS^G12C^ inhibition indirectly increases professional antigen presentation by promoting the activation of APCs accompanied by an increase of CXCR3-binding chemokine expression by DCs. These effects of KRAS^G12C^ inhibition can explain the elevated CD8^+^ T cell recruitment and the increased T cell activation that we observe upon treatment. These characteristics are a prominent feature of “inflamed” TMEs (*27*, *41*), which are more likely to respond to immunotherapy.

KRAS^G12C^ inhibition alleviates immunosuppressive mechanisms and enhances the infiltration and activation of cytotoxic T cells, accompanied by an increase in checkpoint molecule expression, such as PD1 and LAG-3, even in a very immunosuppressive model like the 3LL ΔNRAS tumors. This TME could be considered optimal for the addition of ICB inhibitors to potentiate a T cell–dependent immune response (*42*). However, the combination of KRAS^G12C^ inhibition with anti-PD1 was only synergistic in the immunogenic tumor model (KPAR^G12C^), but not in the two models that were intrinsically resistant to ICB, one “cold” tumor model lacking neoantigens (KPB6^G12C^) and one “T cell excluded” model that evades antitumor immunity by down-regulating MHC and recruiting immunosuppressive myeloid cells (3LL ΔNRAS). While we cannot rule out a beneficial effect of the combination in all tumors with immune refractory TMEs, as our models certainly do not cover the whole spectrum of immunogenicity observed in NSCLC patients, it will be of utmost importance to identify which patients can benefit from the addition of anti-PD1 inhibitors to KRAS^G12C^ inhibitors and to investigate additional therapeutic strategies for the remaining patients. Our mouse models offer the opportunity for future investigation on additional combinatorial therapies, as the therapeutic approach could differ depending on the mechanism of immune evasion. “Cold” tumor–bearing patients may benefit from the addition of drugs aimed to increase antigen load, such as chemotherapy, radiotherapy, epigenetic modulators, or STING agonists (*43*), whereas targeting immunosuppressive cells could be a valid therapeutic strategy for T cell excluded tumors. KRAS^G12C^ inhibitors can already decrease some myeloid immunosuppressive populations; however, treatment consistently results in an increase in the infiltration of T_regs_, which inhibit cytotoxic T cell activity and might represent an alternative target for combination therapy (*44*).

Several preclinical studies, including this one, have demonstrated that combinations of KRAS^G12C^ inhibitors with anti-PD1 can result in therapeutic benefit in immunogenic mouse cancer models (*8*, *19*). On the basis of these data, a number of different clinical trials are underway testing combinations of KRAS^G12C^ inhibitors and PD1 pathway ICB, such as KRYSTAL-1, KRYSTAL-7, CodeBreak 100, and CodeBreak 101, with results eagerly awaited. With these and other clinical trials already running, there are still open questions that need to be addressed to set up the basis for patient stratification. Our findings are particularly relevant for those patients with highly immune refractory TMEs as they could benefit instead from other combination strategies. While it is likely that the ongoing trials of combinations of KRAS^G12C^ inhibitors with immunotherapies will be beneficial for a subset of KRAS-mutant NSCLC patients, this study has highlighted the need for additional treatment strategies in highly immune refractory patients. In particular, it should be noted that most of these combination clinical trials, with the exception of KRYSTAL-7, do not exclude prior treatment with immunotherapy, and are therefore likely to be enriched with patients whose tumors show either intrinsic or acquired resistance to ICB. Extrapolating from the preclinical studies reported here, these patients may be less likely to benefit from combinations of KRAS^G12C^ inhibitors with immunotherapies.

While KRAS^G12C^ inhibitors have only recently been approved for clinical use, MEK inhibitors, targeting the MAPK pathway downstream of KRAS, have been used for some time and can result in similar tumor cell–intrinsic immunomodulatory changes (*45*) and in some cases have shown to ameliorate antitumor immunity (*46*–*49*). However, the positive effects in the TME caused by the tumor cell–intrinsic changes can be reduced by the detrimental effects of MEK inhibition on immune cells (*8*). Moreover, although combinations of inhibitors targeting MAPK pathway plus anti-PD1 can improve clinical outcomes, they do so at the expense of increased toxicities (*50*, *51*). In contrast, KRAS^G12C^ inhibitors offer the unique ability to improve antitumor immunity via a myriad of mechanisms discussed above while not affecting MAPK signaling in nontumor cells, including those involved in the antitumor immune response. Consequently, unlike other targeted therapies that do not specifically target oncogenic mutant proteins, KRAS^G12C^ inhibitors have the potential to achieve long-term survival that is dependent on the activation of antitumor immune responses in immunogenic tumors. The tumor cell–specific activity of KRAS^G12C^ inhibitors provides an unprecedented opportunity to investigate combinations of multiple therapeutic approaches without producing excessive toxicity profiles. Several clinical trials are testing combinations of KRAS^G12C^ inhibitors with other targeted therapies, including MEK inhibitors (*29*). It will be important to validate that the beneficial effects upon the TME are not lost when these two drug classes are combined. With that in mind, it is possible the “vertical” combinations of KRAS^G12C^ inhibitors with SHP2 inhibitors upstream or CDK4/6 inhibitors downstream may be more promising, as both these drug types have also been shown to produce positive immunomodulatory effects (*20*, *52*, *53*).

## MATERIALS AND METHODS

### Study design

The objective of this study was to examine non–tumor cell–intrinsic effects of KRAS^G12C^ inhibitors. We performed controlled (nonblinded) laboratory experiments using cancer cell lines to examine the effects of KRAS^G12C^ inhibitors on gene and protein expression and coculture systems with immune cells to assess indirect effects of the drug treatment on different cell populations. For all in vitro experiments, a minimum of two biological replicates (independent experiments) were acquired.

We also used transplantable murine lung cancer models to assess the effects of KRAS^G12C^ inhibitors in nonblinded randomized studies (alone or in combination with ICB) on mouse survival. Endpoints were predefined and not modified throughout the duration of the study, and mice whose cause of death could not be attributed to lung tumors were excluded. Other in vivo experiments aimed to investigate the TME, by combining RNA, flow cytometry, and IMC data. Sample size was chosen empirically based on results of previous studies, and no data points, including outliers, were excluded from these analyses.

### In vivo tumor studies

All studies were performed under a UK Home Office–approved project license and in accordance with institutional welfare guidelines. For subcutaneous tumor injection, cells were mixed 1:1 with Geltrex matrix (Thermo Fisher Scientific) and 400,000 3LL ΔNRAS or 150,000 KPAR^G12C^ cells were injected in a total volume of 100 μl subcutaneously into one flank of 8-week-old C57BL/6 mice. Tumor growth was followed twice a week by caliper measurements, and tumors were left to grow not larger than 1.5 cm in diameter following a UK Home Office–approved project license.

For orthotopic growth, 10^6^ 3LL ΔNRAS or 150,000 KPAR^G12C^ cells were injected in phosphate-buffered saline (PBS) in a total volume of 100 μl in the tail vein of 8-week-old C57BL/6 mice. Mouse weight was monitored regularly as a measure of tumor growth, and mice were sacrificed if weight loss was over 15% as per the UK Home Office–approved project license. Tumor burden was also assessed by regular computed tomography (CT) scanning of the lungs. Briefly, mice were anesthetized by inhalation of isoflurane and scanned using the Quantum GX2 micro-CT imaging system (PerkinElmer) at a 50-μm isotropic pixel size. Serial lung images were reconstructed, and tumor volumes were subsequently analyzed using Analyse (AnalyzeDirect). For therapeutic experiments, mice were treated daily via oral gavage with MRTX1257 (50 mg/kg; Mirati Therapeutics), MRTX849 (50 mg/kg; MedChemExpress), or 10% Captisol (Ligand) in 50 mM citrate buffer (pH 5.0) as vehicle control.

For ICB treatments, mice were administered anti-PD1 (10 mg/kg; clone RMP1-14, Bio X Cell), anti–PD-L1 (10 mg/kg; clone 10F.9G2, Bio X Cell), and/or anti–LAG-3 (10 mg/kg; clone C9B7W, Bio X Cell) or isotype control (10 mg/kg IgG2b and 5 mg/kg Syrian hamster IgG2) dissolved in PBS at a dose of 4 μl/g mouse intraperitoneally twice a week for a total of four doses.

### Cell lines

NCI-H23 and NCI-H358 were obtained from the Francis Crick Institute Cell Services Facility. 3LL ΔNRAS were generated as previously described (*14*). KRAS^G12V-ER^ pneumocytes were generated as previously described (*14*). KPAR-KRAS^G12C^ and KPB6-KRAS^G12C^ were generated as previously described (*25*). CT26-KRAS^G12C^ were obtained from Mirati Therapeutics (*19*). MutuDC cells were provided by C. Reis e Sousa. KPAR-KRAS^G12C^ were maintained in Dulbecco’s modified Eagle’s medium and MutuDC in Iscove Modified Dulbecco media (IMDM). The rest of the cell lines were cultured in RPMI. Medium was supplemented with 10% fetal calf serum, 4 mM l-glutamine (Sigma-Aldrich), penicillin (100 U/ml), and streptomycin (100 mg/ml; Sigma-Aldrich). Cell lines were tested for mycoplasma and authenticated by short-tandem repeat DNA profiling by the Francis Crick Institute Cell Services Facility. Cells were allowed to grow for not more than 20 subculture passages.

### In vitro drug treatments

Cells were plated at an appropriate density and left to grow for at least 24 hours before drug treatment. Drugs were administered in fresh medium, and samples were collected at indicated time points for downstream analysis. Trametinib (10 nM), GDC0941 (500 nM), everolimus (100 nM), ruxolitinib (500 nM), and decitabine (250 nM) were obtained from Selleckchem. IFNAR blocking antibody (20 mg/ml) was obtained from Bio X Cell. 4-OHT (500 nM) was obtained from Sigma-Aldrich. ARS-1620 (2 μM) was a gift from Araxes Pharma LLC. MRTX1257 (100 nM) was a gift from Mirati Therapeutics. Unless otherwise stated, concentrations used for in vitro experiments are indicated in brackets. Human and mouse recombinant IFNα/β/γ (all from BioLegend) were used at a concentration of 100 ng/ml.

### In vitro viability assay

For viability assays, the CellTiter-Blue assay (Promega) was used. Cells were grown in 96-well plates and treated appropriately for 72 hours. At the end of the experiment, 5 μl of the CellTiter-Blue reagent was added to each well and the reaction was incubated for 90 min in the incubator at 37°C. Fluorescence was subsequently measured using an EnVision plate reader (PerkinElmer) with excitation/emission wavelengths of 560/590 nm.

### Immunoblotting

Cells were lysed using 10X Cell Lysis Buffer [Cell Signaling Technology (CST)], supplemented with EDTA-free protease inhibitor cocktail tablets (Roche), 1 mM phenylmethylsulfonyl fluoride, and 25 mM NaF. Protein (15 to 20 mg) was diluted in NuPAGE LDS Sample Buffer (4×, Thermo Fisher Scientific), and samples were loaded onto NuPAGE 4 to 12% bis-tris protein gels (Thermo Fisher Scientific). Protein transfer to polyvinylidene difluoride membranes was performed using the Trans-Blot Turbo Transfer System (Bio-Rad) or standard manual transferring techniques. For antibody detection, horseradish peroxidase (HRP)–conjugated antibodies were used (GE Healthcare) and data were developed using Amersham Imager 600 (GE Healthcare) or standard film techniques. Immunoblot quantification was performed using ImageJ software (National Institutes of Health).

Antibodies directed against phospho-ERK (extracellular signal–regulated kinase) (T202/Y204, no. 9101), ERK (no. 9107), phospho-AKT (S473, no. 9271), AKT (no. 2920), phospho-S6 (S235/236, no. 2211), S6 (no. 2317), phospho-STAT1 (T701, no. 9167), STAT1 (no. 9172), and STAT2 (no. 4594) were obtained from CST. Pan-RAS antibody was obtained from Merck Millipore (MABS195), vinculin (V9131) was obtained from Sigma-Aldrich, and c-MYC was obtained (ab39688) from Abcam.

### RAS pulldown assay

Active Ras was measured using the Ras Activation Assay Kit from Millipore following the manufacturer’s instructions. Briefly, cells were lysed in Mg^2+^ Lysis Buffer (MLB; 5% NP-40, 750 mM NaCl, 125 mM Hepes, 50 mM MgCl_2_, 5 mM EDTA, and 10% glycerol) containing protease inhibitors. Five hundred micrograms of protein was incubated with RAF-RBD–containing agarose beads and rotated for 75 min at 4°C. Pulled-down protein was then analyzed by immunoblotting using 20 μg of non–bead-incubated protein to normalize for total Ras levels.

### CRISPR-Cas9 knockout

Phosphorylated and annealed *Ccl2*-targeting [sgRNA (single guide RNA) 1 (3′-gRNA-5′): ACACGTGGATGTCTCCAGCCG and sgRNA 2 (5′-gRNA-3′): GCAAGATGATCCCAATGAGT] or Ifngr2-targeting sgRNAs (3′-gRNA-5′: AGGGAACCTCACTTCCAAGT) were cloned into target vector px458-pSpCas9(BB)-2A-GFP (Addgene no. 48138) or px459-pSpCas9(BB)-2A-Puro (Addgene no. 62988), respectively. 3LL ΔNRAS or KPAR^G12C^ cells were transfected using Lipofectamine (Thermo Fisher Scientific) with the px458 vector and fluorescence-activated cell sorting (FACS)–sorted for GFP expression or selected using puromycin treatment. Cells were then single cell–cloned before knockout screening via Sanger sequencing and protein analysis via enzyme-linked immunosorbent assay (ELISA) or FACS.

### Small interfering RNA transfection

siGENOME small interfering RNAs (siRNAs) against mouse Stat1, Stat2, or Myc (Dharmacon) were transfected at a final concentration of 50 nM using DharmaFECT 4 transfection reagent (Dharmacon). The transfection complex was incubated for 20 to 40 min before adding dropwise to freshly seeded cells. As a control, cells were either mock-transfected (no siRNA) or transfected with a siGENOME RISC-free control (Dharmacon).

### Quantitative reverse transcription PCR

RNA was extracted using the RNeasy Mini Kit (QIAGEN) following the manufacturer’s instructions. For in vivo tumor samples, tumors were individually isolated from the lungs, lysed, and homogenized using QIAshredder (QIAGEN) following the manufacturer’s instructions before RNA extraction. SuperScript II Reverse Transcriptase (Thermo Fisher Scientific) was then used to generate cDNA. qPCR was performed using SYBR Green FAST Master Mix (Applied Biosystems).

For a list of primers used, see Table 1. Gene expression changes relative to the housekeeping genes were calculated using the ΔΔCT method.

**Table 1.** List of qPCR primers. Hs, human; Mm, mouse. Primers from QIAGEN have unknown sequence.

**Gene**	**Species**	**Forward**	**Reverse**	**Catalog no.**
*ACTB*	Hs	NA	NA	QT00095431
*B2m*	Mm	TCTCACTGACCGGCCTGTAT	ATTTCAATGTGAGGCGGGTG	
*Ccl2*	Mm	CACTCACCTGCTGCTACTCA	GCTTGGTGACAAAAACTACAGC	
*Cd274*	Mm	CGCCACAGCGAATGATGTTT	AGGATGTGTTGCAGGCAGTT	
*Cd8*	Mm	GAACTGGGAAACAAACCGGC	ATAGCACCCCAGGAAGCCTA	
*Ciita*	Mm	CAAGGATCTTCCTGCCATCCG	CCAGGTGTTGCAGAGAAGAGA	
*Cxcl1*	Mm	ACTCAAGAATGGTCGCGAGG	GTGCCATCAGAGCAGTCTGT	
*Cxcl10*	Mm	AATGAGGGCCATAGGGAAGC	AGCCATCCACTGGGTAAAGG	
*Cxcl11*	Mm	GAAGGTCACAGCCATAGCCC	CTCTGCCATTTTGACGGCTT	
*Cxcl2*	Mm	AGGGCGGTCAAAAAGTTTGC	CAGGTACGATCCAGGCTTCC	
*Cxcl9*	Mm	CCAAGCCCCAATTGCAACAAA	GTCCGGATCTAGGCAGGTTT	
*Dusp6*	Mm	GAGCCAAAACCTGTCCCAGT	GTGACAGAGCGGCTGATACC	
*Foxp3*	Mm	CAGAGAGAAGTGGTGCAGTCTC	GGCTACGATGCAGCAAGAGC	
*GAPDH*	Hs	NA	NA	QT00079247
*Gapdh*	Mm	CAAGCTCATTTCCTGGTATGACA	GGATAGGGCCTCTCTTGCTC	
*Gzma*	Mm	CTGTGCTGGCGCTTTGATTG	TGAGTGAGCCCCAAGAATGAA	
*Gzmb*	Mm	NA	NA	QT00114590
*H2-d1*	Mm	NA	NA	QT01657761
*H2-k1*	Mm	GACCGTTGCTGTTCTGGTTG	TCACGCTAGAGAATGAGGGTCA	
*HSP90*	Hs	AGATTCCACTAACCGACGCC	TGCTCTTTGCTCTCACCAGT	
*Hsp90*	Mm	AGATTCCACTAACCGACGCC	TGCTCTTTGCTCTCACCAGT	
*Ifng*	Mm	ACAGCAAGGCGAAAAAGGATG	TGGTGGACCACTCGGATGA	
*Ifngr1*	Mm	TGCCTGGGCCAGAGTTAAAG	TACGAGGACGGAGAGCTGTT	
*Ifngr2*	Mm	TCACCTTCCAGCAATGACCC	ACCTATGCCAAGAGCCATCG	
*IRF1*	Hs	CCAAATCCCGGGGCTCATC	CTGCTTTGTATCGGCCTGTG	
*Irf1*	Mm	GACCCTGGCTAGAGATGCAG	CTCCGGAACAGACAGGCATC	
*Irf2*	Mm	AATTCCAATACGATACCAGGGCT	GAGCGGAGCATCCTTTTCCA	
*Irf7*	Mm	GCGTACCCTGGAAGCATTTC	GCACAGCGGAAGTTGGTCT	
*IRF9*	Hs	TCCTCCAGAGCCAGACTACT	CAATCCAGGCTTTGCACCTG	
*Irf9*	Mm	GCCGAGTGGTGGGTAAGAC	GCAAAGGCGCTGAACAAAGAG	
*MYC*	Hs	TACAACACCCGAGCAAGGAC	TTCTCCTCCTCGTCGCAGTA	
*Myc*	Mm	CCGGGGAGGGAATTTTTGTCT	GAGGGGCATCGTCGTGG	
*Ncr1*	Mm	CTTGCACCTACCGACCCTAC	TTGTGTGATCCCAGAAGGCG	
*Pdcd1*	Mm	ACCCTGGTCATTCACTTGGG	CATTTGCTCCCTCTGACACTG	
*Prf1*	Mm	TGGAGGTTTTTGTACCAGGC	TAGCCAATTTTGCAGCTGAG	
*Sdha*	Mm	TCGACAGGGGAATGGTTTGG	TCATACTCATCGACCCGCAC	
*Stat1*	Mm	AAGTCTGGCAGCTGAGTTCC	TCTTCGGTGACAATGAGAGGC	
*STAT2*	Hs	ACCATTCTGGACATGGCTGG	CTCCGACTCACAAAGCCCAT	
*Stat2*	Mm	CCCTGGTCGACCTATTGCTG	CAAGAACTTTGCTCCAGCCG	
*Stat3*	Mm	ACGAAAGTCAGGTTGCTGGT	TGTGTTCGTGCCCAGAATGT	

### RNA sequencing

RNA was extracted as indicated above. RNA quality was measured using the 2100 Bioanalyzer (Agilent). Libraries were prepared using the KAPA Hyper Prep Kit (Roche) and sequenced (sequencing read length, 75 base pairs) in an Illumina HiSeq 4000 system. Briefly, reads were aligned using the relevant reference genome (mouse Ensembl GRCm38—release 89 for 3LL and human Ensembl GRCh38—release 38 for human cell lines). For data analysis, the R package DESeq2 was used and gene set enrichment analysis was performed following gene sets available from MSigDB (Broad Institute).

### Whole-exome sequencing and neoantigen prediction

DNA was extracted from cells using the QuickExtract DNA Extraction Solution (Lucigen), and sequencing was performed with 110× coverage using 100–base pair paired end read lengths. DNA library prep was performed using a SureSelectXT reagent kit (Agilent), and genomic DNA (gDNA) was sequenced using an Illumina HiSeq system.

Sequencing reads were aligned to the *Mus musculus* reference genome (mouse Ensembl GRCm38—release 89). For mutation calling, DNA from WT C57BL/6 mice was taken as a reference and analyzed using the Mutect algorithm developed by the Broad Institute. Whole-exome sequencing data of nonsynonymous single-nucleotide polymorphism (SNP)–containing genes (in .vcf format) were combined with RNA-seq data of expressed genes [TPM (Transcripts Per Kilobase Million) > 0]. Peptide sequences for obtained variants were converted using the SeqTailor tool from Rockefeller University (http://shiva.rockefeller.edu/SeqTailor/), by selecting the mouse reference genome and a window size of 12 amino acids on both sides of the variant. MHC binding prediction was performed using the IEDB 2.22 prediction method (http://tools.iedb.org/mhci/).

### Ex vivo immune cell culture and transwell assay

Femurs and tibias from C57BL/6 mice were dissected and flushed using ice-cold PBS using 21-gauge needles. Flushed cells were centrifuged and filtered through a 45-μm mesh, and monocytes were magnetically isolated using the Monocyte Isolation Kit (BM, mouse) from Miltenyi as per the manufacturer’s instructions.

Cell migration was quantified in duplicate using 24-well Transwell inserts (6.5 μm) with polycarbonate filters (5 μm pore size) (Corning Costar, Acton, MA). Monocytes (0.5 × 10^6^ in 100 μl of RPMI) were added to the upper chamber of the insert. The lower chamber contained 600 μl of RPMI 1640 medium or filtered conditioned medium from tumor cells. The plates were incubated at 37°C in 5% CO_2_ for 1.5 hours, and cells that had migrated into the lower chamber were harvested and counted using flow cytometry.

### Cytokine assays

Medium from cells was harvested and used in the Human Cytokine Array Kit (R&D Systems), as per the manufacturer’s instructions. For detection of CCL2, CXCL9, and CXCL10, Human CCL2/MCP-1 DuoSet ELISA, Mouse CCL2/JE/MCP-1 DuoSet ELISA, Mouse CXCL9/MIG DuoSet ELISA, and Mouse CXCL10/IP-10 DuoSet ELISA kits (from R&D Systems) were used, following the manufacturer’s instructions.

### Immunohistochemistry

Tumor-bearing lungs were fixed in 10% NBF (Neutral buffered formalin) for 24 hours followed by 70% ethanol. Fixed tissue was embedded in paraffin wax. Tissue sections were stained with hematoxylin and eosin, using standard methods. For immunohistochemistry staining, tissue sections were boiled in sodium citrate buffer (pH 6.0) for 15 min and incubated with the following antibodies for 1 hour: anti-Foxp3 (D6O8R, CST) and anti-CD8 (4SM15, Thermo Fisher Scientific). Primary antibodies were detected using biotinylated secondary antibodies and detected by HRP/DAB (3,3′-Diaminobenzidine). Slides were imaged using a Leica Zeiss AxioScan.Z1 slide scanner.

### Flow cytometry

Mice were culled using schedule 1 methods, and lungs were dissected (one spleen was also dissected to use as single stain control). Tumors were dissected from the lungs and cut into small pieces before incubating in digestion solution (1 mg/ml collagenase type I and 50 U/ml deoxyribonuclease in Hanks’ balanced salt solution buffer) at 37°C for 30 min. After homogenization, samples were filtered through a 70-μm cell strainer, erythrocytes were shocked using ACK lysing buffer (Life Technologies), and samples were refiltered through 70-μm cell strainers. After washes in PBS, samples were stained with fixable viability dye eFluor780 (BD Horizon) for 30 min at 4°C. Samples were washed three times in FACS buffer (2 mM EDTA and 0.5% bovine serum albumin in PBS, pH 7.2) and stained using appropriate antibody mixes or single stain controls (spleen or OneComp eBeads from Thermo Fisher Scientific). After staining, samples were fixed in fix/lyse (Thermo Fisher Scientific) or FixPerm solution (Thermo Fisher Scientific) if intracellular staining was needed. Samples were then either stained with an intracellular antibody or washed and analyzed using a FACSymphony analyzer (BD). Data were analyzed using FlowJo software v10 (LLC).

For FACS analysis in vitro, cells were harvested with trypsin, filtered, and washed in FACS buffer before appropriate antibody treatment. For intracellular cytokine staining, cells were treated with brefeldin A (BD GolgiPlug) for 6 hours before harvesting. Cells were permeabilized using the FixPerm (Thermo Fisher Scientific) solution before staining. Samples were run in LSRII or LSRFortessa (BD), and FlowJo software v10 (LLC) was used to analyze the data. For a list of antibodies used, see Table 2.

**Table 2.** List of FACS antibodies.

**Target**	**Fluorophore**	**Clone**	**Source**	**Catalog no.**
H-2L^d^/H-2D^b^	PE	28-14-8	BioLegend	114507
H2-Kb	AF647	AF6-88.5	BioLegend	116512
CD45	PerCP	30-F11	BioLegend	103130
CD3	FITC	17A2	BioLegend	100204
gdTCR	BV605	GL3	BioLegend	118129
CD4	BUV737	GK1.5	BD Biosciences	564298
CD8	BUV395	53-6.7	BD Biosciences	563786
Foxp3	eFluor660	FJK-16s	eBioscience	50-5773-82
CD44	BV421	IM7	BioLegend	103039
CD62L	BV711	MEL-14	BioLegend	104445
CD69	BV605	JES5-16E3	BioLegend	104530
PD1	BV785	29F.1A12	BioLegend	135225
LAG-3	PE-Cy7	eBioC9B7W	eBioscience	25-2231-82
NKp46	BV421	29A1.4	BioLegend	137612
CD49b	AF488	DX5	BioLegend	108913
CD19	PE	6D5	BioLegend	115507
B220/CD45R	BV605	RA3-6B2	BioLegend	103244
CD11c	BUV395	HL3	BD Biosciences	564080
CD11b	BUV737	M1/70	BD Biosciences	564443
Ly6G	BV711	1A8	BioLegend	127643
Ly6C	BV785	HK1.4	BioLegend	128041
PD-L1	PE	MIH5	eBioscience	12-5982-81
F4/80	BV785	EMR1	BioLegend	123141
CD24	BV605	M1/69	BioLegend	101827
CD103	BV421	M290	BD Biosciences	562771
CD64	PE-Cy7	X54-5/7.1	BioLegend	139314
CD206	BV711	C068C2	BioLegend	141727
TIM3	PE	RMT3-23	BioLegend	119703
CD86	BV785	GL-1	BioLegend	105043
MHCII	FITC	M5/114.15.2	BioLegend	107605
CXCL9	PE	MIG-2F5.5	BioLegend	515603

### Imaging mass cytometry

Tissue processing and antibody staining was performed as described in detail in (*21*). In short, 5-μm cryosections of fresh frozen lungs were fixed (Image-iT Fixative Solution, Thermo Fisher Scientific) and stained with the antibody panel listed in Table 3 and Cell-ID Intercalator-Ir (Fluidigm). Scanning of the (dried) slides was done with the Hyperion Imaging Mass Cytometer (Fluidigm). Images are available at the Figshare repository https://doi.org/10.25418/crick.19590259.

**Table 3.** List of IMC antibodies.

**Metal**	**Target**	**Clone**	**Source**	**Catalog no.**
89Y	CD45	30-F11	Fluidigm	3089005B
141Pr	aSMA	1A4	Fluidigm	3141017D
142Nd	MHCcII	M5/114.15.2	BioLegend	107637*
144Nd	MHCcI	28-14-8	Fluidigm	3144016C
146Nd	F480	(CI:A3-1)	Bio-Rad	MCA497GA*
147Sm	CD68	FA-11	BioLegend	137002*
150Nd	CD44	IM7	Fluidigm	3150018B
152Sm	CD3e	145-2C11	Fluidigm	3152004B
153Eu	PDL1	10F.9G2	Fluidigm	3153016B
158Gd	Foxp3	FJK-16s	Fluidigm	3158003A
161Dy	CD103	AF1990G	R&D Systems	AF1990*
165Ho	TIGIT	4D4/mTIGIT	BioLegend	156102*
166Er	PD1	29F.1A12	BioLegend	135202*
167Er	NKp46	29A1.4	Fluidigm	3167008B
168Er	CD8a	53-6.7	Fluidigm	3168003B
169Tm	CD4	RM4-5	BioLegend	100561*
170Er	CXCL9	MIG-2F5.5	BioLegend	515602*
171Yb	Granzyme B	GB11	Fluidigm	3171002C
172Yb	Cleaved caspase 3	5A1E	Fluidigm	3172027D
173Yb	Ki67	16A8	BioLegend	652402*
174Yb	LAG-3	C9B7W	Fluidigm	3174019C
176Yb	B220	RA3-6B2	Fluidigm	3176002B
209Bi	CD11c	N418	Fluidigm	3209005B

*Antibodies conjugated in house using MaxPar antibody labeling kits (Fluidigm).

Image processing was performed with the previously described 1-pixel expansion single-cell segmentation pipeline using imcyto (nf-core/imcyto). The resulting single-cell data were clustered with Phenograph and subsequently annotated to the different cell types (table S1).

### Statistical analysis

For most experiments, data were compared using unpaired or paired two-tailed Student’s *t* tests or analysis of variance (ANOVA) if more than two experimental groups were examined. In mouse tumor analysis, the Mann-Whitney *U* test was used for volume comparison. To compare read counts of individual genes in mRNA-seq datasets of two groups, Wald test was used with a Benjamini and Hochberg correction with a false discovery rate *Q* value of 5% to obtain adjusted *P* values (statistical analysis was performed by Crick Bioinformatics Facility). To compare two survival curves, the Mantel-Cox log-rank test was used. Statistical analyses were performed in Prism 7 (GraphPad Software) or in RStudio. Significance is presented as *P < 0.05, **P < 0.01, ***P < 0.001, and ****P < 0.0001.

## Notes

View/request a protocol for this paper from *Bio-protocol*.
