# Supplementary material for: Therapeutic KRASG12C inhibition drives effective interferon-mediated antitumor immunity in immunogenic lung cancers
Source: Sci Adv. 2022 Jul 20;8(29):eabm8780. doi: 10.1126/sciadv.abm8780 (PMC9299537; doi:10.1126/sciadv.abm8780)
Supplement: Supplementary file 1 — Figs. S1 to S7 [file sciadv.abm8780_sm.pdf]

Science Advances
AAAS

Supplementary Materials for

Therapeutic $KRAS^{G12C}$ inhibition drives effective interferon-mediated antitumor immunity in immunogenic lung cancers

Edurne Mugarza *et al.*

Corresponding author: Julian Downward, julian.downward@crick.ac.uk; Miriam Molina-Arcas, miriam.molina@crick.ac.uk

Sci. Adv. **8**, eabm8780 (2022)
DOI: 10.1126/sciadv.abm8780

The PDF file includes:

Figs. S1 to S7
Legend for table S1

Other Supplementary Material for this manuscript includes the following:

Table S1

SUPPLEMENTAL MATERIAL

Supplementary figure 1 (related to figure 1)

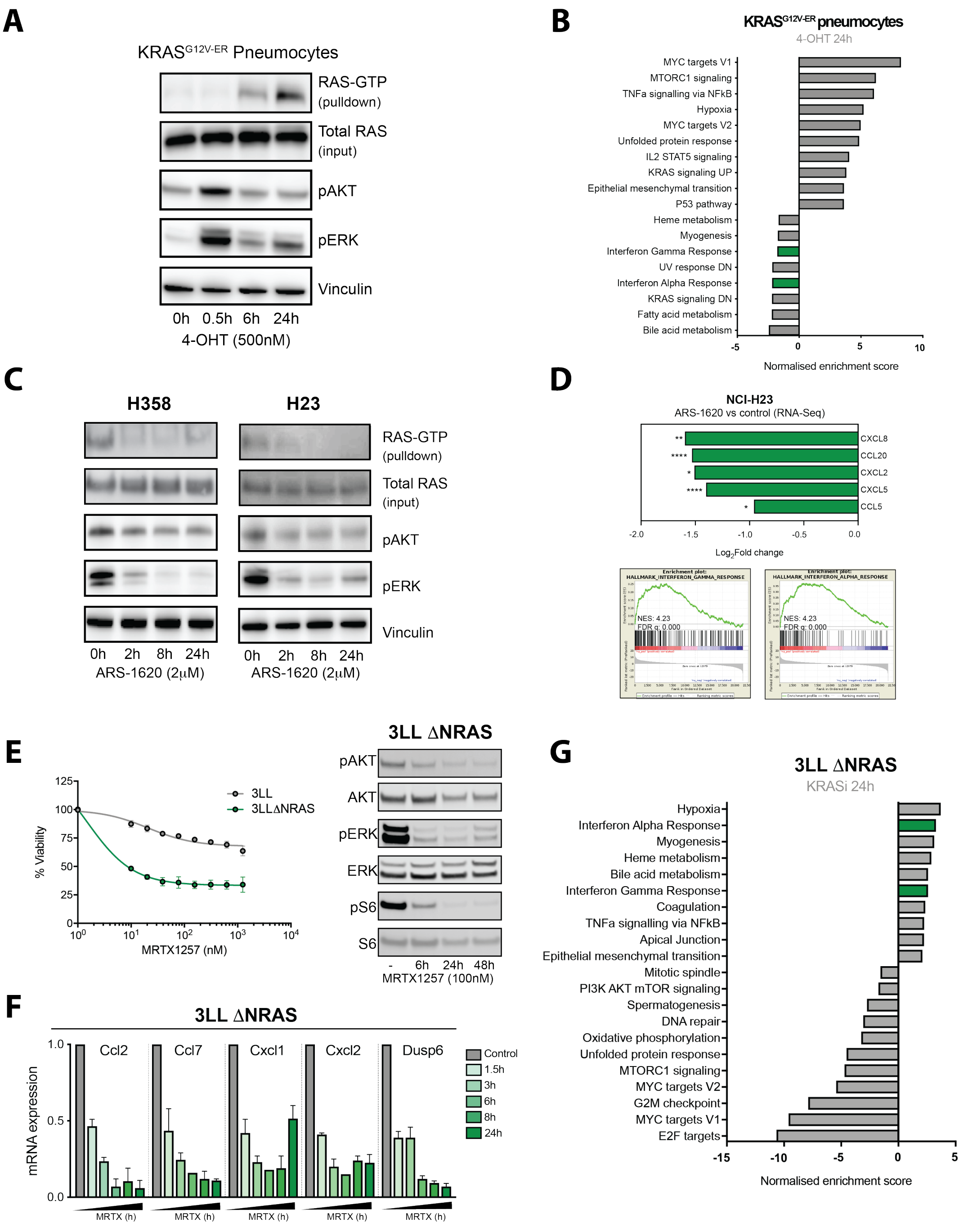

Fig. S1. KRAS-dependent regulation of secreted factors in human and murine cell lines. (A) Time course of $KRAS^{G12V\text{-}ER}$ pneumocytes treated with 500nM 4-OHT showing increased active KRAS (KRAS-GTP) and downstream pathway activation. (B) Summary of most up- and down-regulated pathways (MSigDB Hallmarks, FDR q<0.05) in 4-OHT treated $KRAS^{G12V\text{-}ER}$ pneumocytes. (C) Time course of human $KRAS^{G12C}$ lung cancer cell lines treated with 2μM ARS-1620 showing decreased active KRAS (KRAS-GTP) and downstream pathway activation. (D) Top: Log_2Fold change of selected cytokine genes from RNA-Seq data in ARS-1620 (2μM, 24h, p adjusted value) treated NCI-H23 cells versus DMSO control. Bottom: MSigDB Hallmarks GSEA plots of IFNα and IFNγ pathway genes in ARS-1620 treated versus control samples. (E) Left: viability assay comparing parental 3LL and CRISPR-edited 3LL ΔNRAS cells treated with increasing concentrations of MRTX1257 for 72h (n=2 independent experiments, mean±SEM). Right: Time course of 3LL ΔNRAS cells treated with 100nM MRTX1257 showing downstream pathway inhibition. (F) Time course analysis of MRTX1257-treated 3LL ΔNRAS cells showing mRNA expression of cytokines and Dusp6 as a control for KRAS inhibition ($2^{-\Delta\Delta CT}$, normalised to control sample for all genes, n=2, mean+SEM). (G) Summary of most up- and down-regulated pathways (MSigDB Hallmarks, FDR q<0.05) in ARS-1620-treated 3LL ΔNRAS cells.

Supplementary figure 2 (related to figure 2)

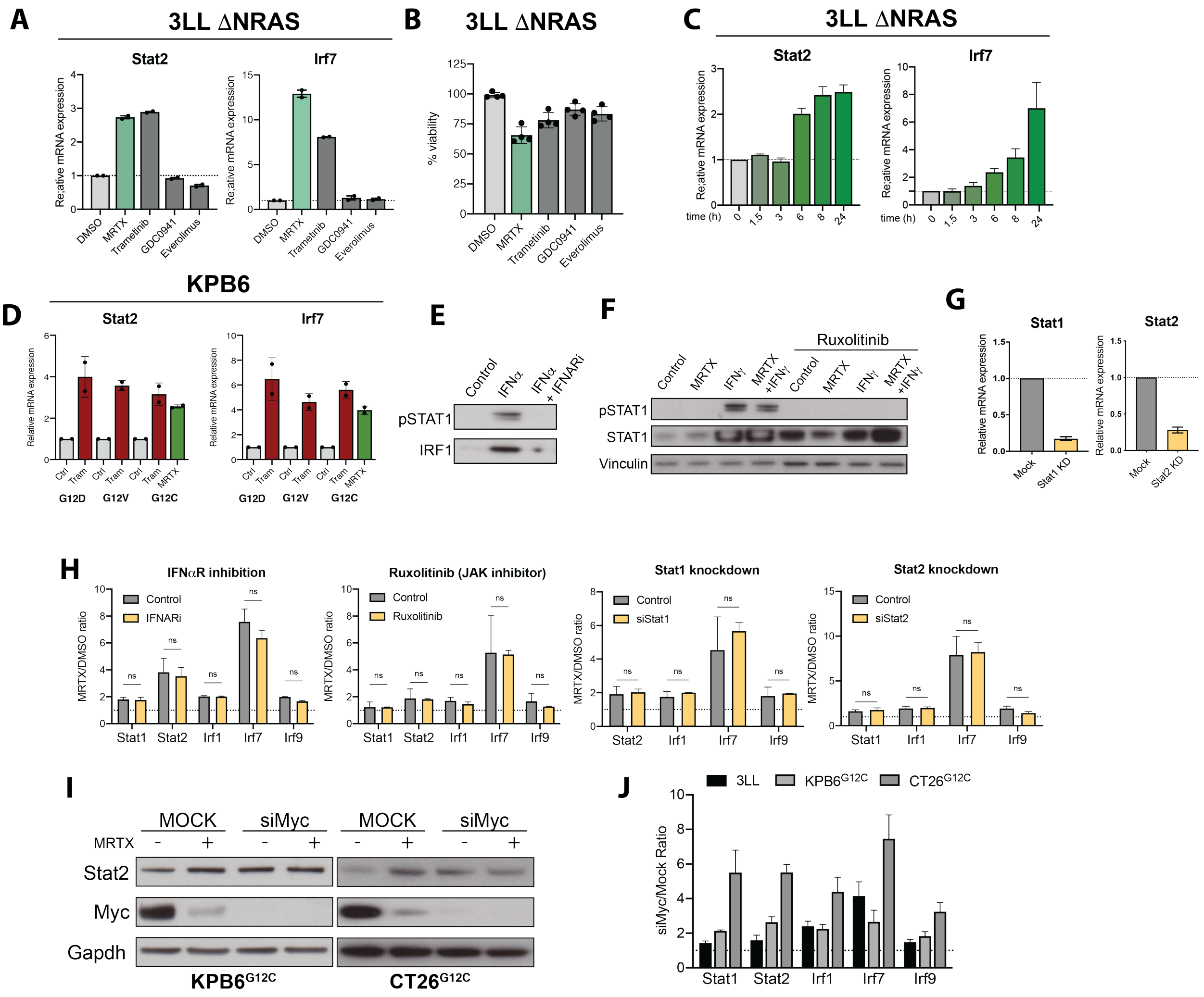

Fig. S2. Mechanism of KRAS-dependent regulation of IFN genes.

(A) Expression of IFN-induced genes *Stat2* and *Irf7* in 3LL ΔNRAS cells treated with DMSO control, MRTX1257 (100nM), MEK inhibitor trametinib (10nM), PI3K inhibitor GDC0941 (500nM) or mTOR inhibitor everolimus (100nM) for 24h ($2^{-\Delta\Delta CT}$, normalised to control sample, n=2, mean±SEM). (B) Viability of 3LL ΔNRAS cells treated with drugs as in (A) for 24h (n=4). (C) 100nM MRTX1257 treatment time course of 3LL ΔNRAS cells ($2^{-\Delta\Delta CT}$, normalised to control sample, n=3, mean+SEM). (D) mRNA expression of Stat2 and Irf7 after trametinib (10nM, 24h) treatment of isogenic KPB6 cell lines with differing KRAS G12 mutations and comparison with MRTX1257 (100nM) treatment in KPB6^{G12C} cell line. ($2^{-\Delta\Delta CT}$, normalised to control sample for each cell line, n=2, mean±SEM) (E) Western blot showing loss of IFN sensitivity after IFNαR (20mg/ml, 24h) blocking antibody treatment (100ng/ml IFNα, 24h) of 3LL ΔNRAS cells. (F) Western blot showing loss of IFN sensitivity by 1μM ruxolitinib (100ng/ml IFNγ, 24h). (G) Knockdown efficiency of siStat1 (48h, left) and siStat2 (48h, right) measured by qPCR ($2^{-\Delta\Delta CT}$,

normalised to control sample, n=3). (H) Comparison of the ratio of IFN-induced gene expression in MRTX- versus DMSO-treated 3LL ΔNRAS in control cells and cells treated with an anti-IFNaR antibody treatment (20mg/ml), 1μM JAK1/2 inhibitor Ruxolitinib treatment, Stat1 or Stat2 knockdown (24h, $2^{-\Delta\Delta CT}$, n=3, paired t test). (I) Western blot of KPB6^{G12C} and CT26^{G12C} cells showing *Myc* knockdown and *Stat2* increase after treatment with MRTX, Myc siRNA, or both. (J) Ratio of Myc siRNA versus mock control treatment expression of IFN-induced genes in 3LL ΔNRAS (n=3), KPB6^{G12C} (n=4) and CT26^{G12C} (n=3) cells.

Supplementary figure 3 (related to figure 3)

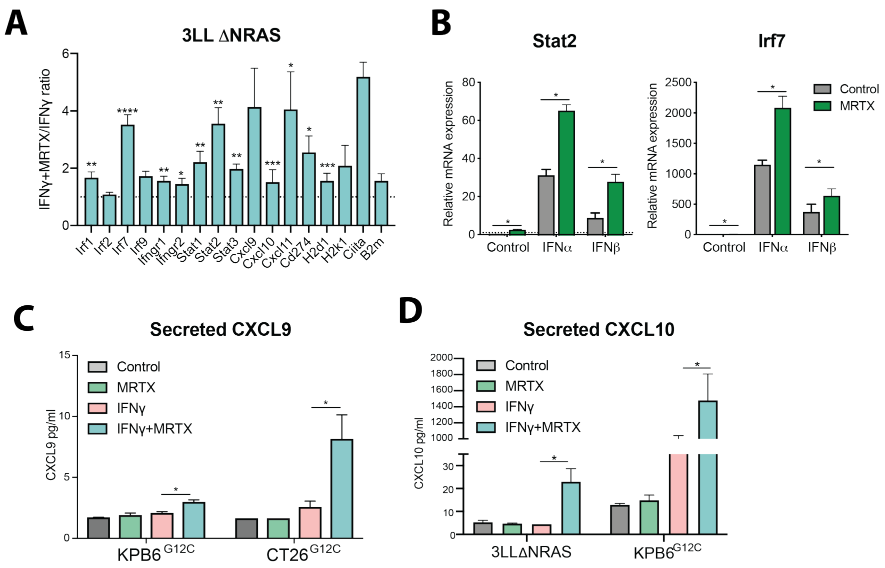

Fig. S3. KRAS-driven augmentation of type I and II IFN treatment response.

(A) Summary of all IFN-stimulated genes (ISG) examined, showing the ratio of IFNγ plus MRTX1257 (100nM) versus IFNγ (100ng/ml) alone (24h treatment, $2^{-\Delta\Delta CT}$, normalised to IFNγ-treated sample for all genes, n at least 3, mean+SEM). (B) mRNA expression of IFN-induced genes *Stat2* and *Irf7* after treatment of 3LL ΔNRAS cells with recombinant IFNα/β (100ng/ml) and/or MRTX for 24h ($2^{-\Delta\Delta CT}$, normalised to control, n=3, paired t test, mean+SEM). (C) Concentration of CXCL9 secreted to the cell culture supernatant by CT26^{G12C} and KPB6^{G12C} cells after treatment with MRTX, IFNγ or both (normalised to control, n=2 for KPB6^{G12C}, n=3 for CT26^{G12C}, mean+SEM, paired t test). (D) Concentration of CXCL10 secreted to the cell culture supernatant by 3LL ΔNRAS and KPB6^{G12C} cells after treatment with MRTX, IFNγ or both (normalised to control, n=3, mean+SEM, paired t test).

Supplementary figure 4 (related to figure 4)

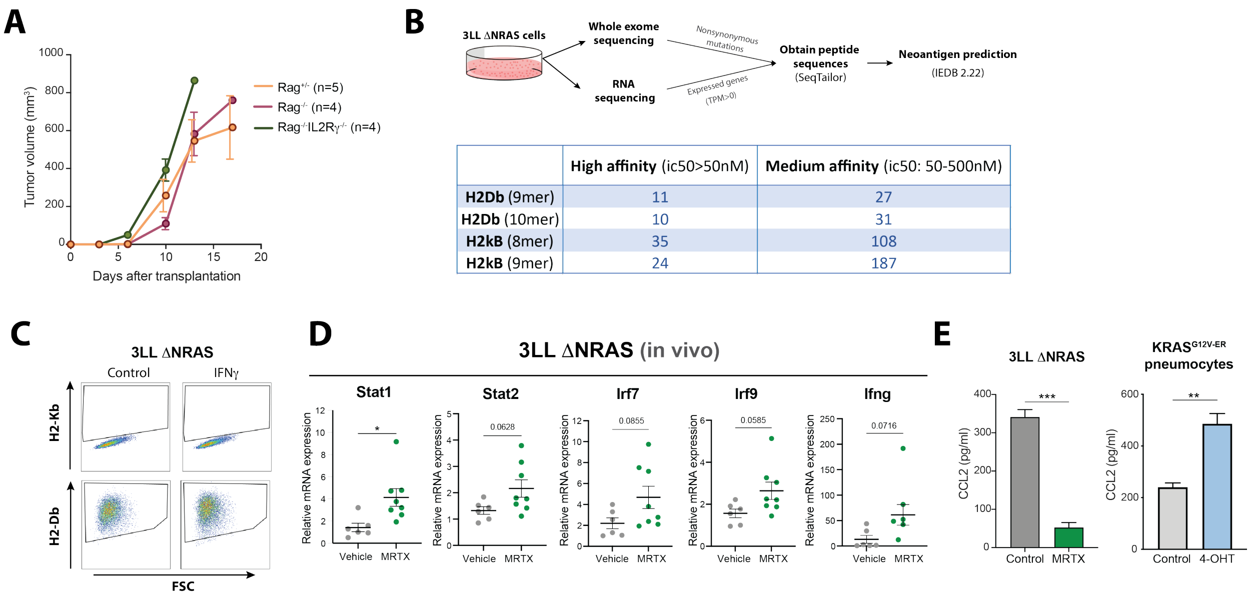

	High affinity (ic50>50nM)	Medium affinity (ic50: 50-500nM)
**H2Db** (9mer)	11	27
**H2Db** (10mer)	10	31
**H2kB** (8mer)	35	108
**H2kB** (9mer)	24	187

Fig. S4. In vivo characterisation of 3LL ΔNRAS tumours and the effects of KRAS^{G12C} inhibition on myeloid cells. (A) Growth comparison of subcutaneously implanted 3LL ΔNRAS tumours in Rag1$^{+/-}$, Rag1$^{-/-}$ and Rag1$^{-/-}$IL2Rγ$^{-/-}$ mice. (B) Above: summary of in silico analysis merging whole exome sequencing and RNA-Seq data to obtain predicted neoantigens. Below: number of predicted high and medium affinity neoantigens obtained for each C57Bl/6 MHC allele for different sized peptides. (C) Flow cytometric analysis of 3LL ΔNRAS cells in vitro showing lack of basal and IFNγ-induced (100ng/ml, 24h) expression of surface H2-Kb, and intact H2-Db expression. (D) qPCR analysis of IFN-induced genes in 3LL ΔNRAS lung tumours ($2^{-\Delta\Delta CT}$, vehicle n=6, MRTX n=8, unpaired t test, mean±SEM). (E) Concentration of secreted CCL2 as measured by ELISA in medium from either control or 4-OHT-treated KRAS$^{G12V-ER}$ pneumocytes (n=3, Mean+SEM, unpaired t test).

Supplementary figure 5 (related to figure 5 and 6)

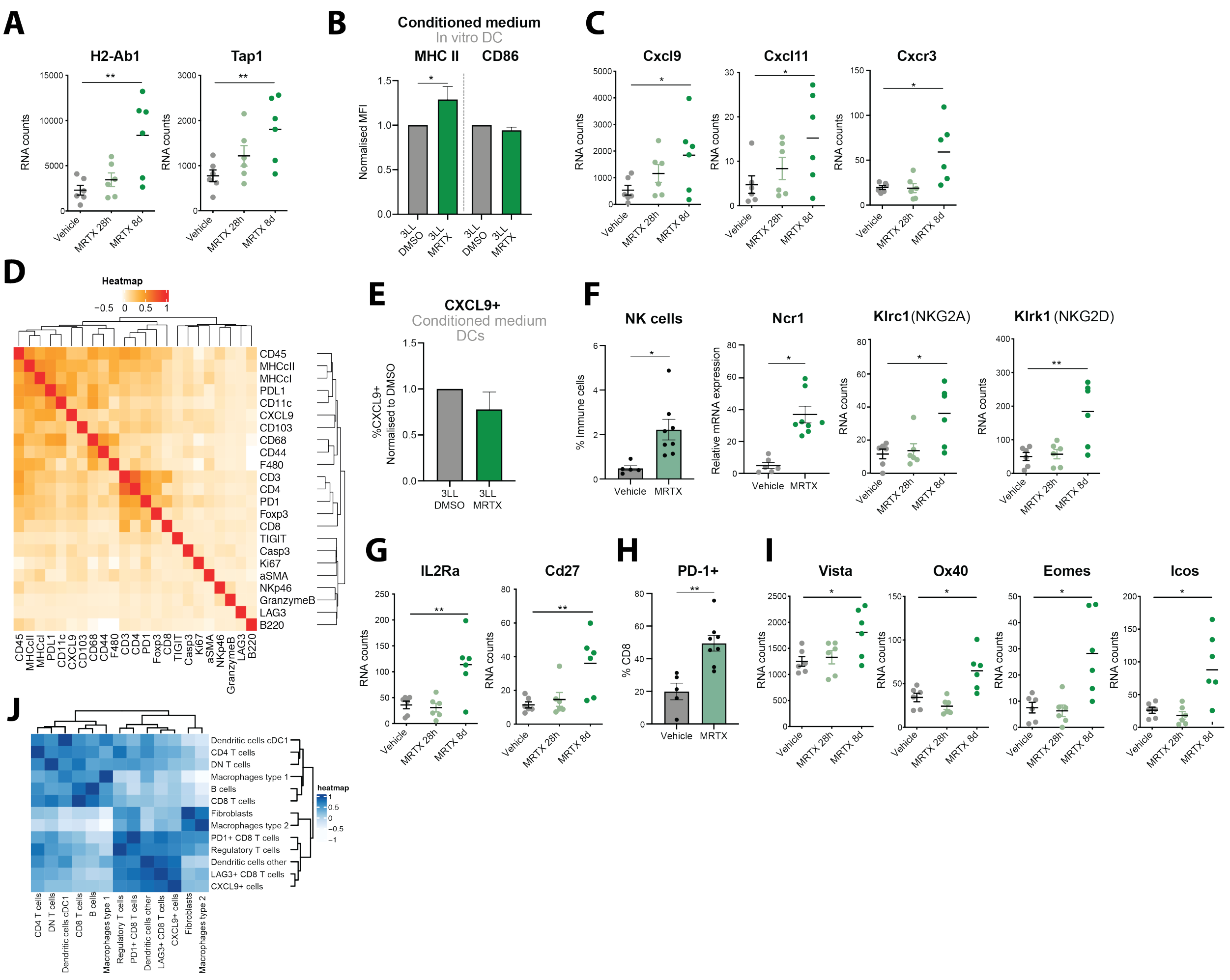

Fig. S5. KRAS^{G12C} effects on antigen presentation, T cell infiltration and activation in vivo. (A) mRNA counts showing increased expression of antigen presentation genes from RNA-Seq analysis of 3LL ΔNRAS lung tumours treated with vehicle or 50mg/kg MRTX1257 for 28h or 8d (FDR p adjusted value, n=6 tumours per group). (B) Normalised mean fluorescence intensity of MHC II and CD86 on DCs cultured overnight under filtered conditioned medium from either DMSO-treated or MRTX-treated (48h) 3LL ΔNRAS cells (n=3, mean+SEM, 2-way ANOVA). (C) mRNA counts for T cell chemoattractant and receptor-encoding genes, analysed as in (A). (D) Pearson correlation matrix of markers expressed at single cell level as measured by IMC. (E) Normalised percentage of CXCL9+ DCs after overnight incubation as in (B), analysed as in (B). (F) NK cell data summary after one week of MRTX1257 treatment in vivo. Left: increased NK cell infiltration in tumours as measured by flow cytometry (pre-gated as CD45+ CD19- NKp46+ CD49b+, n=5 for vehicle, n=8 for MRTX-treated, unpaired t test). Middle: qPCR analysis for NK cell marker Ncr1 (6 samples per group, unpaired t test). Right: mRNA count data for NK cell markers *Klrc1* and *Klrk1*, analysed as in (A). (G) mRNA counts for T cell activation genes *IL2Rα*

and *Cd27*, analysed as in (A). (H) Percentage of PD-1+ CD8+ T cells measured by flow cytometry (vehicle n=5, MRTX n=8, unpaired t test, mean±SEM). (I) mRNA counts showing increased expression of T cell exhaustion genes from RNA-Seq of lung 3LL ∆NRAS tumours treated with vehicle or MRTX, analysed as in (A). (J) Pearson correlation matrix based on cell proportions present within the tumour and interface domain of MRTX-treated tumours measured by IMC.

Supplementary figure 6 (related to figure 7)

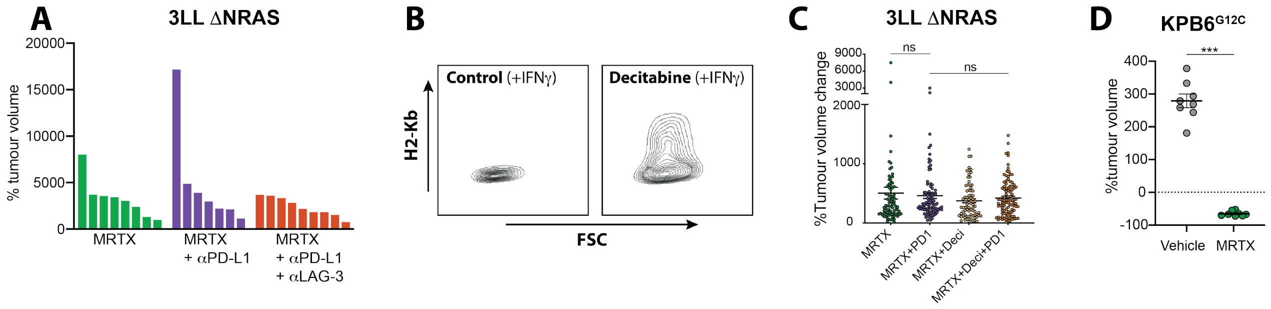

Fig. S6. Lack of combinatorial effects of KRAS^{G12C} inhibition and ICB in immune refractory tumours. (A) Waterfall plot showing tumour volume change of 3LL ΔNRAS lung tumours treated with MRTX only (n=8 mice), 50mg/kg MRTX1257 and 10mg/kg anti-PD-L1 antibody (n=7 mice) or 50mg/kg MRTX1257, 10mg/kg anti-PD-L1 and 10mg/kg anti-LAG3 antibody combination (n=9) displaying no combinatorial effect. (B) H2-kB expression measured by flow cytometry of IFNg (100ng/ml) treated 3LL ΔNRAS cells after decitabine (5'Aza-2'-deoxycytidine, 250nM, 24h) treatment. (C) Tumour volume change of 3LL ΔNRAS lung tumours treated with MRTX1257 (50mg/kg, n=9 mice), MRTX+anti-PD-1 (10mg/kg, n=10 mice), MRTX+Deci (0.3mg/kg, n=9 mice) or the triple combination (7 day treatment, 2-way ANOVA). (D) Tumour volume change of KPB6^{G12C} tumours after one week treatment with 50mg/kg MRTX1257 showing marked regression (n=8 mice per group, Mann-Whitney analysis).

Supplementary figure 7 (related to figure 8 and 9)

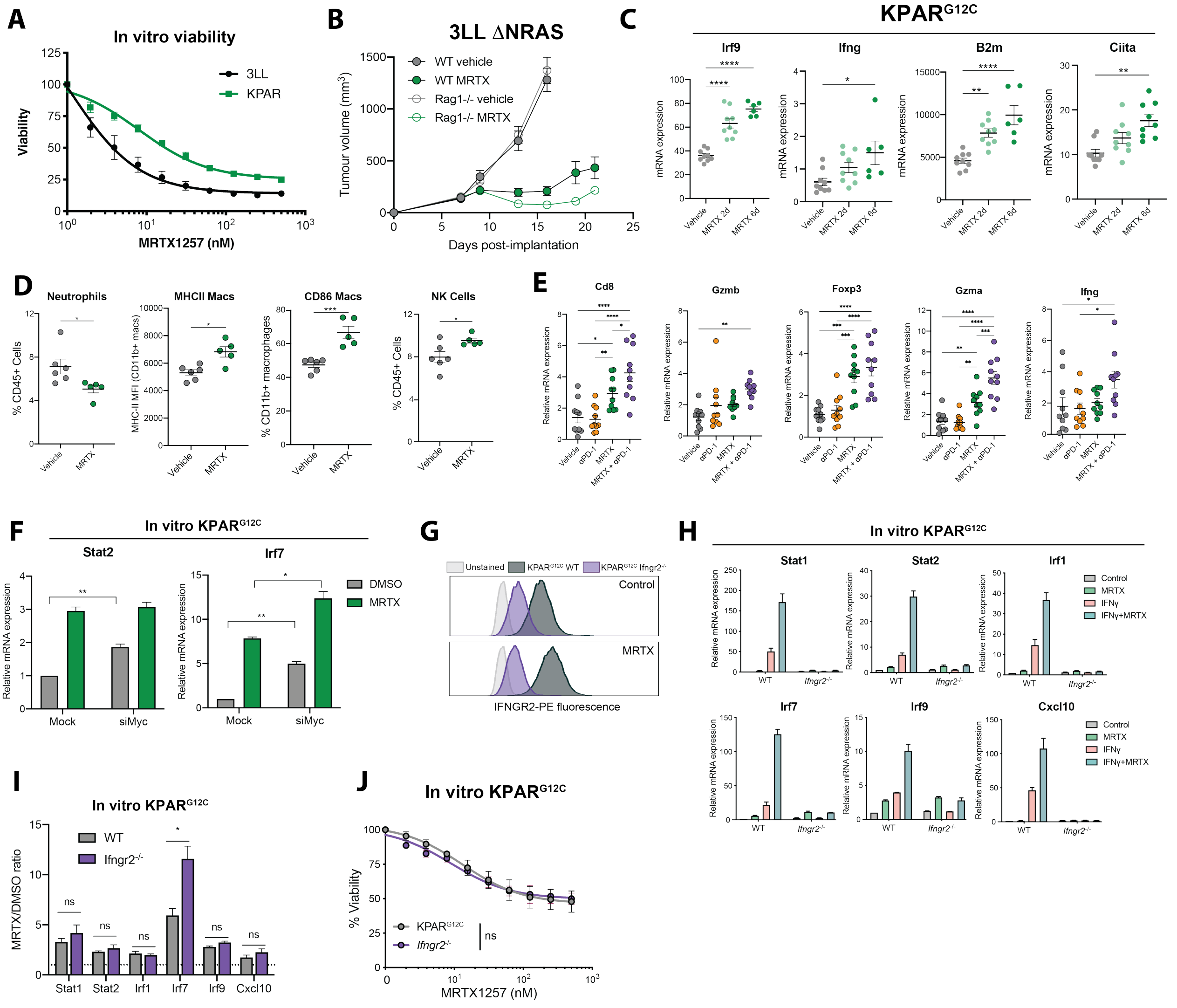

Fig. S7. Effects of KRAS^{G12C} inhibition in immunogenic tumours and role of IFN signalling. (A) Viability data for 3LL ΔNRAS and KPAR^{G12C} cells treated in vitro with increasing concentrations of MRTX1257 for 72h. (B) Subcutaneous 3LL ΔNRAS tumour growth in C57Bl/6 WT mice treated with vehicle (n=6 mice) or MRTX1257 50mg/kg (n=7 mice) and *Rag1*$^{-/-}$ mice treated with vehicle (n=9 mice) or MRTX1257 (n=10 mice). (C) qPCR analysis of IFN-induced and antigen presentation genes in KPAR^{G12C} tumours treated with vehicle (n=9 tumours) or 50mg/kg MRTX849 for two (n=9 tumours) or 6 days (n=6 tumours, one way ANOVA). (D) Flow cytometry data from MRTX849 treated (4 days) KPAR^{G12C} lung tumours. Neutrophils are gated as live CD45+ CD11b+ Ly6C+ Ly6G+, macrophages as live CD45+ CD11b+ CD24- CD64+ and NK cells as Live CD45+ CD19- NKp46+ CD49b+ (control n=6 mice, MRTX n=5 mice, unpaired t test). (E) qPCR analysis of immune markers on KPAR^{G12C} lung tumours treated for 5 days with 50mg/kg MRTX849 and/or 10mg/kg anti-PD-1, n=10 mice per group (each dot represents a

tumour, ANOVA multiple comparisons test). (F) qPCR analysis of IFN-induced genes $KPAR^{G12C}$ cells treated with MRTX1257, Myc siRNA or both ($2^{-\Delta\Delta CT}$, normalised to control sample for all genes, n=3, paired t tests siMyc versus Mock, mean+SEM). (G) Flow cytometry analysis of surface IFNGR2 expression on $KPAR^{G12C}$ or *Ifngr2*$^{-/-}$ cells in vitro, treated with DMSO or 100nM MRTX1257 for 24h. (H) In vitro qPCR analysis of $KPAR^{G12C}$ WT and *Ifngr2*$^{-/-}$ cells treated with 100nM MRTX1257, 100ng/ml IFNγ or both for 24h ($2^{-\Delta\Delta CT}$, paired t test, n=3, mean+SEM). (I) Same data as in (H), showing the MRTX/DMSO ratio of expression of IFN-related genes in $KPAR^{G12C}$ WT vs *Ifngr2*$^{-/-}$ cells (unpaired t test). (J) In vitro viability of WT and *Ifngr2*$^{-/-}$ $KPAR^{G12C}$ cells treated with a range of doses of MRTX1257 for 72h (n=3, mean±SEM).

Supplementary table 1. IMC single cell data

Single cell data, obtained by segmentation and clustering, of the imaging mass cytometry image dataset deposited on Figshare (https://doi.org/10.25418/crick.19590259).

Each row provides the data for a single cell.

Column description:

- "ROI_name" gives the name of the image file as deposited on Figshare.
- "MouseID", code for the mouse from which the tumour was originally taken.
- "treatment" lists whether a tumour was treated with Vehicle or MRTX.
- "MI_" denotes mean intensity per cell for the markers listed.
- "cluster" the number of cluster assigned by Phenograph and refined supervised gating.
- "clustername" the cell type that was manually assigned based on expression profile.
- "Location_Center_X/Y" represent the coordinates of the centre of each cell in the image.
- "dist_" lists the distances for every cell in the image to the nearest of the cell type as called in the name of the column.
